# Functional Connectivity at Rest and During Cognitive Processing in Multiple Sclerosis: A Pilot Magnetoencephalography Study and Systematic Review

**DOI:** 10.3390/brainsci16080862

**Published:** 2026-08-14

**Authors:** Anza B. Memon, Anas Z. Nourelden, Mouhamad Hammami, Basil Memon, Ahmed Hashem Fathallah, Farid Ahmed Badar, Mirela Cerghet, Lonni R. Schultz, Susan M. Bowyer

**Affiliations:** 1 John D. Dingell, VAMC, Detroit, MI 48201, USA; eg6818@wayne.edu; 2 Department of Neurology, School of Medicine, Wayne State University, Detroit, MI 48201, USA; mcerghe1@hfhs.org (M.C.); smbowyer@gmail.com (S.M.B.); 3 Department of Neurology, Henry Ford Hospital, Detroit, MI 48202, USA; fbadar1@hfhs.org; 4 Department of Physics, Oakland University, Rochester Hills, MI 48309, USA; mhammami@oakland.edu; 5 Department of Psychology, University of Michigan, Ann Arbor, MI 48109, USA; basil.memon@gmail.com; 6 Faculty of Medicine, Minia University, Minia 61522, Egypt; dr.ahmedhashem12@gmail.com; 7 Department of Public Health Sciences, Henry Ford Health, Detroit, MI 48202, USA; lschult1@hfhs.org

**Keywords:** MEG, multiple sclerosis, MS, cognition, systematic review

## Abstract

**Background/Objectives**: Cognitive impairment and neurological dysfunction in Multiple Sclerosis are increasingly understood as consequences of altered brain network connectivity. Magnetoencephalography offers high temporal resolution for investigating functional connectivity patterns, which may underlie various clinical symptoms. This exploratory pilot study aims to characterize preliminary FC patterns at rest and during cognitive processing in MS patients with a high fatigue burden compared with healthy controls using MEG, complemented by a systematic review of existing MEG literature in MS. **Methods**: In this pilot study, MEG was utilized to investigate connectivity in MS patients and HCs. Data were acquired during resting-state and a digitized version of the Symbol Digit Modalities Test. A systematic review was also conducted following PRISMA guidelines to synthesize the current evidence on frequency-specific MEG connectivity in MS. **Results**: MS patients exhibited a connectivity pattern paralleling the directionality of neuronal slowing, described in the spectral power MS literature, during resting-state, characterized by reduced interhemispheric coherence in alpha and beta bands alongside region-specific increases in frontal–parietal and parietal–occipital alpha coherence, reflecting a bidirectional spatial reorganization. Conversely, during the SDMT task, the MS group demonstrated significant compensatory recruitment with increased coherence in theta and gamma bands (specifically in frontal and striatal circuits). The systematic review of 44 MEG studies corroborated these findings, highlighting a consistent trend of frequency-dependent dysconnectivity that aligns with clinical load and disease pathology. **Conclusions**: We found that in MS, network alterations are state-dependent, shifting from reduced connectivity at rest to increased coherence during cognitive effort. These preliminary findings suggest that MEG-based connectivity may serve as a functional neuroimaging biomarker of underlying network reorganization, for monitoring early cognitive disease progression in MS.

## 1. Introduction

Understanding the neurophysiological basis of Multiple Sclerosis (MS) is crucial, as the disease is increasingly recognized as a “disconnection syndrome” [1,2,3]. In this context, neurological deficits arise not only from focal lesions but from the widespread disruption of functional brain networks. Several functional imaging techniques for assessing the impact of MS on cognition are available, including functional magnetic resonance imaging (fMRI), positron emission tomography (PET), and magnetoencephalography (MEG). Numerous studies using these techniques have provided valuable insights into the neural mechanisms underlying cognitive dysfunction in patients with MS [1,2,4,5,6]. fMRI and PET measure secondary neural response from hemodynamics and glucose consumption, while MEG measures the magnetic field associated with actual neuronal activity. Functional brain activity associated with cognition during a modified Symbol Digit Modalities Test (SDMT), a validated measure of cognitive processing speed in MS and other neurodegenerative disorders, which examinees have 90 s to match abstract symbols to paired digits using a reference key, has been assessed with fMRI, revealing no differences in accuracy between patients with MS and healthy controls. Nonetheless, individuals with MS had significantly slower processing speeds than controls [7]. Moreover, fMRI activation during the SDMT was seen in the precentral gyrus and occipital cortex in both groups, but those with MS showed significantly less cerebral activity in the bilateral frontal and parietal regions [7]. Complementing these imaging modalities, pure neurophysiological techniques, such as TMS-EEG, have provided evidence of cortical excitability alterations, functional connectivity disruption, and network-level reorganization in MS, revealing deficits in cortical inhibition and plasticity [8]. Overall, these studies have provided a detailed picture of changes in neuronal activity in patients with MS but fall short of providing the temporal resolution of brain activity processing. To understand the temporal dynamics of underlying brain processes, a brain imaging technique such as MEG becomes necessary.

MEG is a noninvasive functional brain imaging technique that measures the magnetic brain waves arising from neuronal activity during rest or a specific task, and this technique is capable of real-time functional imaging with a spatial resolution down to several millimeters and a temporal resolution of less than 1 ms. A study combining MEG and fMRI observed reduced neuronal and hemodynamic responses during visual stimulation in patients with MS compared to controls. This materialized as a significant reduction in peak gamma power in MS patients compared to control subjects. This reduction could indicate gray matter dysfunction, possibly mediated by GABAergic changes [1]. However, findings regarding gamma oscillations remain inconsistent, particularly when comparing passive stimulation to active cognitive tasks. Also, MEG showed that mental fatigue suppresses activities in the anterior cingulate cortex elicited by mental imagery of physical effort [2]. Another study investigating MEG aimed at identifying the neurophysiological markers of cognitive impairment in patients with MS analyzed global spectral and connectivity, revealing that cognitively impaired patients had higher theta and alpha1 power (8–10 Hz) and lower alpha2 and beta power (10–13 Hz) than patients with no cognitive impairment in several regions of interest [3]. Yet other studies implementing MEG to investigate MS have focused on disrupted networks in the resting state (i.e., theta, alpha, and beta frequency bands) [9,10,11,12,13,14,15,16], leaving a gap in understanding how these connectivity patterns shift during active cognitive challenge.

Despite these insights, the neurophysiological transition between resting-state and active cognitive effort and how it relates to the overall network reorganization in MS remains incompletely characterized. Therefore, this exploratory pilot study aims to investigate preliminary frequency-specific connectivity signatures within four neuronal activity bands during both rest and a cognitive task (SDMT) in MS patients compared with controls. By integrating our experimental results with a comprehensive systematic review, we seek to provide a robust profile of MEG functional connectivity changes in MS that could serve as an objective imaging biomarker for monitoring disease status and progression.

## 2. Materials and Methods

In this pilot study conducted at a single center, we investigated the impact of MS-related fatigue on cognition compared to age- and gender-matched healthy individuals. We used the SDMT, a validated standardized cognitive scale widely used in MS research as a sensitive measure to assess cognitive processing speed and attention; it is scored as the total number of correct substitutions made in 90 s under timed conditions [17,18]. For this study, a digitized version adapted for MEG administration was employed. Additionally, we used MEG to map the underlying brain areas involved in cognitive processes. Patients were evaluated during a resting state and while performing a cognitive task.

### 2.1. Subject Population and Eligibility

Participants were recruited from the Henry Ford Health MS Clinic. All participants underwent a full Informed Consent process and provided written consent to participate in the study. This study was approved by the Henry Ford Health Institutional Review Board [IRB No. 15400].

#### 2.1.1. Inclusion Criteria

Eligible patients were adults (18–60 years old), diagnosed with RRMS or clinically isolated syndrome meeting McDonald’s 2017 diagnostic criteria for MS, with a history of MS-related fatigue; currently taking disease-modifying therapies (DMT); and Kurtzke’s Expanded Disability Status Scale (EDSS) score ≤ 4.0. Patients taking anti-fatigue medications, such as amantadine, modafinil, and methylphenidate, were given a 4-week washout period (based on the medications’ elimination half-lives) before study enrollment [19].

#### 2.1.2. Exclusion Criteria

Patients were excluded if they had used steroids for MS exacerbation within the past 30 days, had a history of severe anxiety, depression, psychiatric disorders, stroke, excessive alcohol use, or traumatic brain injury. Additionally, patients with uncontrolled medical risk factors such as hypertension, hyperlipidemia, and diabetes, which could increase the risk for chronic ischemic microvascular disease and potential cognitive impairment, were also excluded.

### 2.2. Experimental Setups

**Figure 1:**
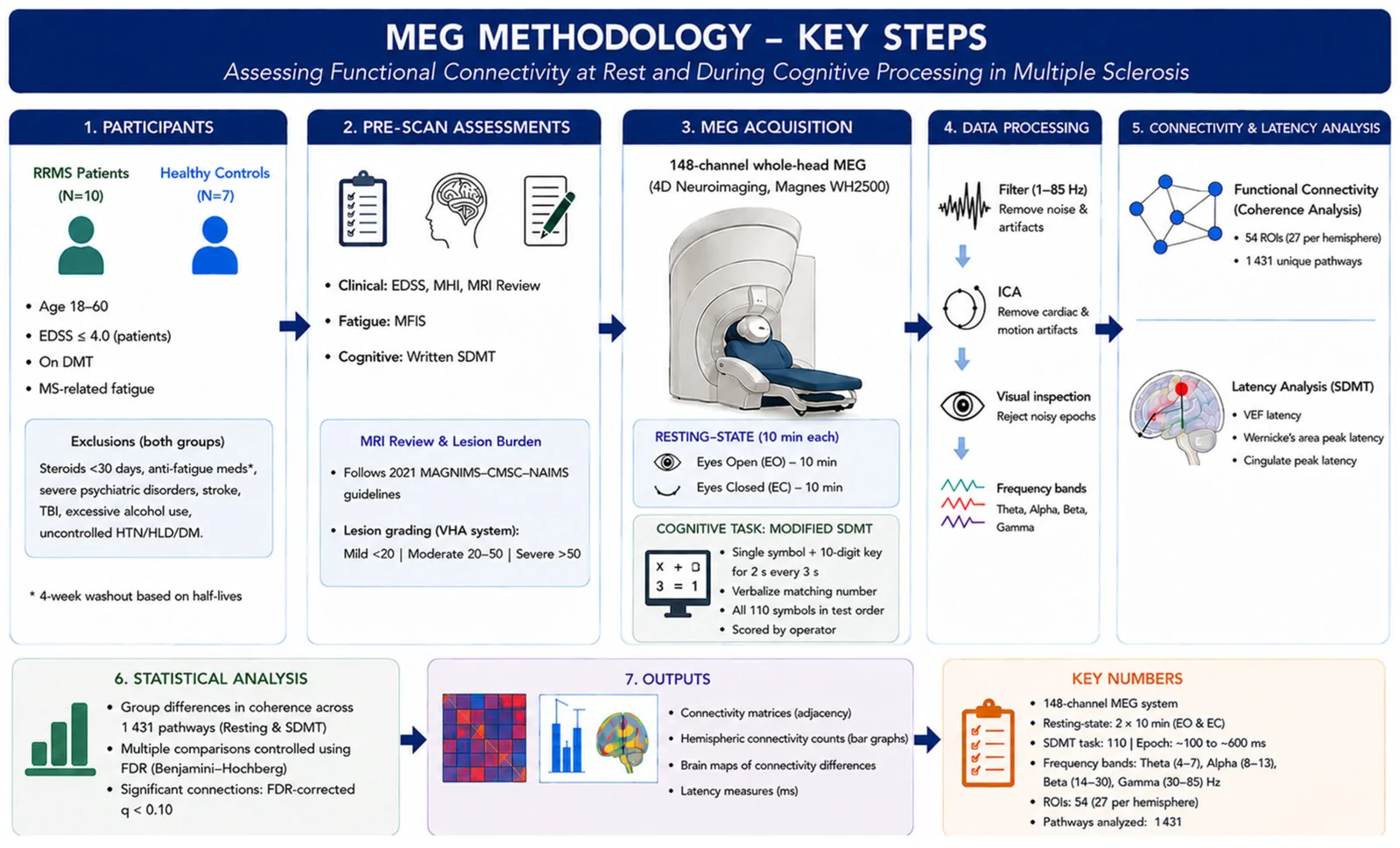
MEG setup steps illustration.

Before MEG sessions, MS patients underwent clinical evaluations, including the EDSS, Mental Health Inventory (MHI), and written SDMT. The Modified Fatigue Impact Scale (MFIS) assessed baseline physical fatigue. Healthy controls also completed the MFIS and SDMT before scanning. During MEG, patients performed a modified SDMT task, adapted from prior fMRI studies, after obtaining required approval for test use [7,20]. Demographics, medical history, disease/fatigue duration, radiographic findings, and disease-modifying therapies (DMTs) were retrieved from EPIC (electronic medical records). Brain and spinal cord MRI images were reviewed to assess disease burden. MRI interpretation followed the 2021 MAGNIMS–CMSC–NAIMS consensus guidelines [21]. Lesion burden was graded using the Veterans Health Administration (VHA) system: mild (<20 lesions), moderate (20–50), or severe (>50) [22]. While this grading approach aligned with MAGNIMS–CMSC–NAIMS criteria, severity stratification was not explicitly recommended by the consensus. A summary of the experimental workflow, including MEG data acquisition, preprocessing steps, and connectivity analysis, is illustrated in Figure 1.

### 2.3. Brain Imaging Measurements and Analyses

#### 2.3.1. Recording

Cortical activity was recorded using a 148-channel whole-head MEG system (Magnes WH2500; 4D Neuroimaging, San Diego, CA, USA) with magnetometer sensors. Participants wore a standard medical gown and removed all metal objects. Five fiducial coils were placed on each subject: three across the forehead and one in front of each ear. These coils were digitized to map scalp locations, and head shapes were recorded for MRI co-registration. Before and after each run, the coils were activated with a low current to detect head movement. Runs were repeated if motion exceeded 5 mm; no participants moved beyond this threshold during scanning. Subjects lay supine with their head positioned inside a helmet-shaped sensor array within a magnetically shielded room. A bowl-shaped head holder minimized motion, and the MEG helmet was secured without obstructing the face. MEG measures functional brain activity, allowing for the analysis of network dynamics and connectivity patterns during cognitive processing.

#### 2.3.2. MEG Data Acquisition and Analysis

During acquisition, data were band-pass filtered 0.1 to 100 Hz and digitally sampled at 508.63 Hz, the standard native sampling rate of the 4D Neuroimaging Magnes WH2500 system, and continuously recorded for later analysis. The total time of MEG testing was approximately 30 min. The resting-state scans consisted of two continuous 10 min acquisitions of spontaneous brain activity (one scan with eyes open and one with eyes closed). The cognitive processing task was the SDMT, which was modified and adapted for MEG, similar to approaches used with fMRI [7,20]. A mirror was placed above the subjects, angled so they could view a projected image on the screen. A single SDMT symbol was visually displayed along with the 10-digit key above it for 2 s every 3 s. Subjects verbalized responses as soon as they identified the correct number to match the displayed symbol. A person outside of the room scored the correct answers. All 110 SDMT symbols from the test were displayed in the same order as in the original test.

#### 2.3.3. MEG Data Processing

Resting-state and SDMT data were processed with an additional 1–85 Hz band-pass filter to optimize artifact rejection and remove noise outside the frequency bands of interest. Independent Component Analysis (ICA) was utilized to isolate and remove cardiac and motion-related artifacts. Additionally, data epochs were reviewed to exclude trials contaminated by excessive noise prior to connectivity analysis. Data were then filtered into four frequency bands: theta (4–7 Hz), alpha (8–13 Hz), beta (14–30 Hz), and gamma (30–85 Hz). The upper gamma boundary of 85 Hz was chosen to capture high-gamma activity (>60 Hz), which has been shown in MEG studies to be functionally relevant to cognitive and working-memory processing, and was accommodated by the 1–85 Hz preprocessing band-pass filter. We note that gamma definitions in the systematic review literature range from 30–70 Hz to 35–80 Hz, and cross-study gamma comparisons should account for this variability. The delta frequency band (0.5–4 Hz) was excluded from the connectivity analysis, aligning with the vast majority of prior MS MEG literature. It was implemented to minimize the contamination of low-frequency non-neural artifacts, such as respiratory variations and subtle head or jaw movements, which are highly pronounced during active verbal tasks like the SDMT. Coherence analysis of the strength of the functional network connectivity across 1431 pathways derived from all unique pairwise combinations of 54 ROIs, determined using MEG TOOLS, a MATLAB-based software program (RRID:SCR_000661; https://www.nitrc.org/projects/meg-tools; Accessed on 3 June 2026) [23,24,25,26]. The SDMT MEG data were averaged based on the trigger recorded every time the image was displayed (*N* = 110 trials). All epochs had a baseline of 100 ms before stimuli onset and 600 ms of data after stimuli onset. These data were then visually inspected to determine the latencies of the visual evoked response and the cognitive peak in Wernicke’s area. Source reconstruction was performed using MR-FOCUSS, a current distribution algorithm that estimates cortical sources from MEG data. Activity was mapped to standard MRI space through co-registration, allowing localization to specific areas, such as Wernicke’s region.

Co-registration enabled precise correspondence between anatomical structures and MEG-identified cortical activation areas, with errors of less than 5 mm. A standard MRI was transformed into the digitized head shape for each patient to compare group brain activations. Each patient’s brain activity was mapped onto the standard MRI with MEG TOOLS.

#### 2.3.4. MEG Coherence Analysis

Synchronization of neuronal activity was quantified by calculating coherence between 27 cortical sites in each hemisphere [24,25]. The functional connectivity between brain areas can be determined by detecting highly coherent regions in MEG data. Coherence was quantified as magnitude-squared coherence, as implemented in MEG TOOLS, following prior validated applications of this pipeline [23,24]. To calculate coherence [24], MEG data were first divided into 80 segments, each containing a 7.5 s data segment. The high amplitude of cortical activity in each segment was imaged onto the standard MRI using MRFOCUSS, a current distribution imaging technique [26]. Using the time sequence of imaged activity, coherence between active cortical model sites was calculated for each data segment and then averaged across the resting state scan. Additionally, connectivity for each cortical model site was quantified using a histogram of the number of sites with which it shared the same level of coherence. Statistical analysis of cortical coherence levels (ranging from 0 to 1) was used to quantify differences in network connectivity between groups. For additional details of MEG coherence imaging, see Lajiness-O’Neill et al. [23]. Because magnitude-squared coherence can be affected by field spread and volume conduction, we computed it in source space after MR-FOCUSS cortical reconstruction rather than at the sensor level, thereby reducing this contamination. However, source reconstruction does not fully eliminate zero-lag spatial leakage, and this remains a limitation. Phase-invariant metrics such as imaginary coherence or the weighted phase-lag index, are recommended for future work. A region-of-interest (ROI) tool implemented in MEG Tools was used to identify 54 brain regions (27 per hemisphere). MEG Tools utilizes a non-linear volumetric transformation of the patient’s brain to convert MEG coordinates into a standard MRI coordinate system [27,28,29]. This enables the ROI tool to access an atlas of Brodmann’s area identifiers and an atlas of cortical structures [28].

#### 2.3.5. Group Differences in MEG Data

For each MEG frequency band (theta, alpha, beta, and gamma) within each condition (resting state and cognitive SDMT), group differences in functional connectivity were assessed by comparing coherence values across all 1431 possible regional pairs. To address the multiple comparisons problem, a False Discovery Rate (FDR) correction was applied using the Benjamini–Hochberg procedure at a threshold of 0.10 to maximize detection of potentially relevant signals [30]. This threshold was selected over the conventional q < 0.05, given the exploratory, hypothesis-generating nature of this pilot study. No prior power calculation was performed, as published effect-size benchmarks for MEG coherence differences in this specific MS-fatigue phenotype were unavailable at study design. Significant connections surviving the FDR-corrected threshold were then aggregated using a z-score computed according to the Efron method to summarize the difference in coherence values between groups. Positive z-scores indicate higher coherence in the MS group and negative z-scores indicate higher coherence in the HC group. The connections were counted and visualized in connectivity matrices and brain maps to represent the frequency-specific signatures for each group.

### 2.4. Additional Statistical Analyses

SAS software, SAS Institute Inc., Cary, NC, USA (version 9.4) was used for the remaining statistical analyses. Continuous data were described as mean ± standard deviation (SD), and categorical data were described as number and percentage. Independent sample *t*-tests and chi-square tests were performed to compare groups. For demographic and clinical score comparisons, a two-tailed *p*-value < 0.05 was considered statistically significant.

### 2.5. Systematic Review

We conducted our review in accordance with the Preferred Reporting Items for Systematic Reviews and Meta-Analyses (PRISMA) guidelines [31]. This systematic review was not prospectively registered; we appended it to complement the imaging study. We systematically searched MEDLINE (via PubMed), Scopus, Web of Science Core Collection, and the Cochrane Library from database inception to November 2025, using a combination of controlled vocabulary (e.g., MeSH/Emtree) and free-text terms related to multiple sclerosis and magnetoencephalography, with the core strategy (“Multiple Sclerosis”) AND (“Magnetoencephalography” OR MEG OR “magneto-encephalography”). The complete search strategies for all databases are provided in Supplementary Table S1. The reference lists of included studies and relevant reviews were manually searched, and forward citation tracking was performed to identify all relevant articles. Eligibility criteria were defined a priori using the PICO framework: population: individuals with multiple sclerosis; exposure: magnetoencephalographic data acquisition; comparator: healthy controls; and outcomes: any reported MEG-derived parameter, including connectivity metrics, amplitude, power, latency, and topographic measures. We included original English-language studies enrolling at least five patients with multiple sclerosis that quantified at least one MEG parameter, encompassing resting-state, evoked, and event-related paradigms, with no date restrictions. Two reviewers independently screened titles and abstracts, assessed full texts for eligibility, and extracted data on study characteristics (year, country, sample size), population descriptors (MS subtype, baseline EDSS), and MEG findings (frequency bands, analytical metrics, resting-state and task-related results or modulation from baseline), with discrepancies resolved by discussion or third-reviewer adjudication. The quality of the studies was assessed using the National Institutes of Health (NIH) quality assessment tools for observational studies, with domains rated as yes/no/cannot determine/not applicable, and an overall risk-of-bias judgment of “good,” “fair,” or “poor” assigned. The completed PRISMA 2020 checklist is provided in Supplementary Table S2.

## 3. Results

### 3.1. Study Population

A total of 17 participants were included: ten MS patients and seven HCs. The mean ± SD age was 46.7 ± 9.6 years for the controls and 40.3 ± 9.2 years for the MS group. Groups did not significantly differ by sex or age. For MS patients, the median EDSS was 2.75 (range, 1.5–3.5), and the median duration of MS was 5.5 years (range, 1–26 years). The MS group had a significantly lower mean paper SDMT score than the control group (43.3 ± 11.2 vs. 57.7 ± 12.9; *p* = 0.03), suggesting potential cognitive deficits; however, MEG SDMT scores did not differ between groups (Table 1). The paper SDMT is timed and self-paced, so it is sensitive to the processing-speed deficit typical of MS. The MEG version instead displayed each symbol for 2 s and scored accuracy rather than speed. Because each item allowed adequate time, accuracy approached ceiling in both groups. MS Patients reported a significantly greater fatigue impact than control patients, and no differences were observed between groups in MEG latency measures of cognitive processing speed, as identified in the evoked MEG data (Table 1).

### 3.2. MEG Resting and SDMT Connectivity Patterns in MS Patients vs. Controls:

The neurophysiological assessment revealed distinct frequency-specific connectivity signatures in patients with MS compared to HCs. Overall, MS patients demonstrated fewer interhemispheric connections during resting-state, particularly in the alpha, and beta bands. However, a significant shift was observed during the SDMT task, with the MS group showing increased connectivity (higher coherence) in the theta and gamma bands, suggesting state-dependent neural reorganization.

During rest, MS patients exhibited altered connectivity across all bands: Theta Band (4–7 Hz): MS patients showed strong regional coherence, with a distribution involving the right hemisphere (131 significant pathways in MS vs. 79 significant pathways in HC) and left hemisphere (117 pathways in MS vs. 60 pathways in HC), while interhemispheric connections were comparable between groups (209 in MS vs. 218 in HC) (Supplementary Figure S1). Alpha Band (8–13 Hz): MS patients showed higher coherence in frontal–frontal, frontal–parietal, fusiform–striatal, and parietal–striatal pairs, whereas controls showed markedly higher coherence in posterior pairs (e.g., temporal–occipital, occipital–occipital, occipital–parietal), consistent with their higher overall interhemispheric connectivity (298 vs. 134) (Supplementary Figure S2). Beta (14–30 Hz) and Gamma Bands (30–85 Hz): HCs generally showed more extensive connectivity counts compared to MS patients (beta: 814 vs. 25 total significant pathways in HC vs. MS; gamma: 530 vs. 365). In the MS group, connections were more localized, particularly in beta, where left-hemisphere connections were markedly fewer (20 in MS vs. 161 in HC) (Supplementary Figures S3 and S4).

The performance of the SDMT triggered a different connectivity profile: Theta Band: During the task, MS patients showed a marked increase in connectivity counts compared to HCs (612 vs. 262 total significant pathways), particularly in the left hemisphere (181 in MS vs. 43 in HC) and temporal–occipital regions (Supplementary Figure S5). Alpha Band: Unlike the resting state, task-related alpha connectivity was more comparable between groups (414 vs. 476 total significant pathways in MS vs. HC), with interhemispheric connections predominating in both (202 in MS, 272 in HC) (Supplementary Figure S6). Beta Band: HCs continued to show higher overall connectivity (553 vs. 214 total significant pathways), but MS patients demonstrated a shift toward increased left-hemisphere coherence during the task compared to rest (67 during SDMT vs. 20 at rest) (Supplementary Figure S7). Gamma Band: During cognitive effort, MS patients exhibited enhanced connectivity (632 vs. 265 total significant pathways), showing a higher count of left-hemispheric connections compared to HCs (183 in MS vs. 46 in HC) (Supplementary Figure S8).

A summary of these MEG-resting and SDMT differences is presented in Table 2. Connectivity differences are shown in Supplementary Figures S1–S8, where red areas indicate the MS group had more connectivity than the control group, whereas green areas indicate the control group had more connectivity. In these images, only differences between the groups are shown, while brain areas that were active in both groups are shown in the histograms and heatmaps.

### 3.3. Systematic Review Results

#### 3.3.1. Included Studies Characteristics and Their Participant Demographics

**Figure 2:**
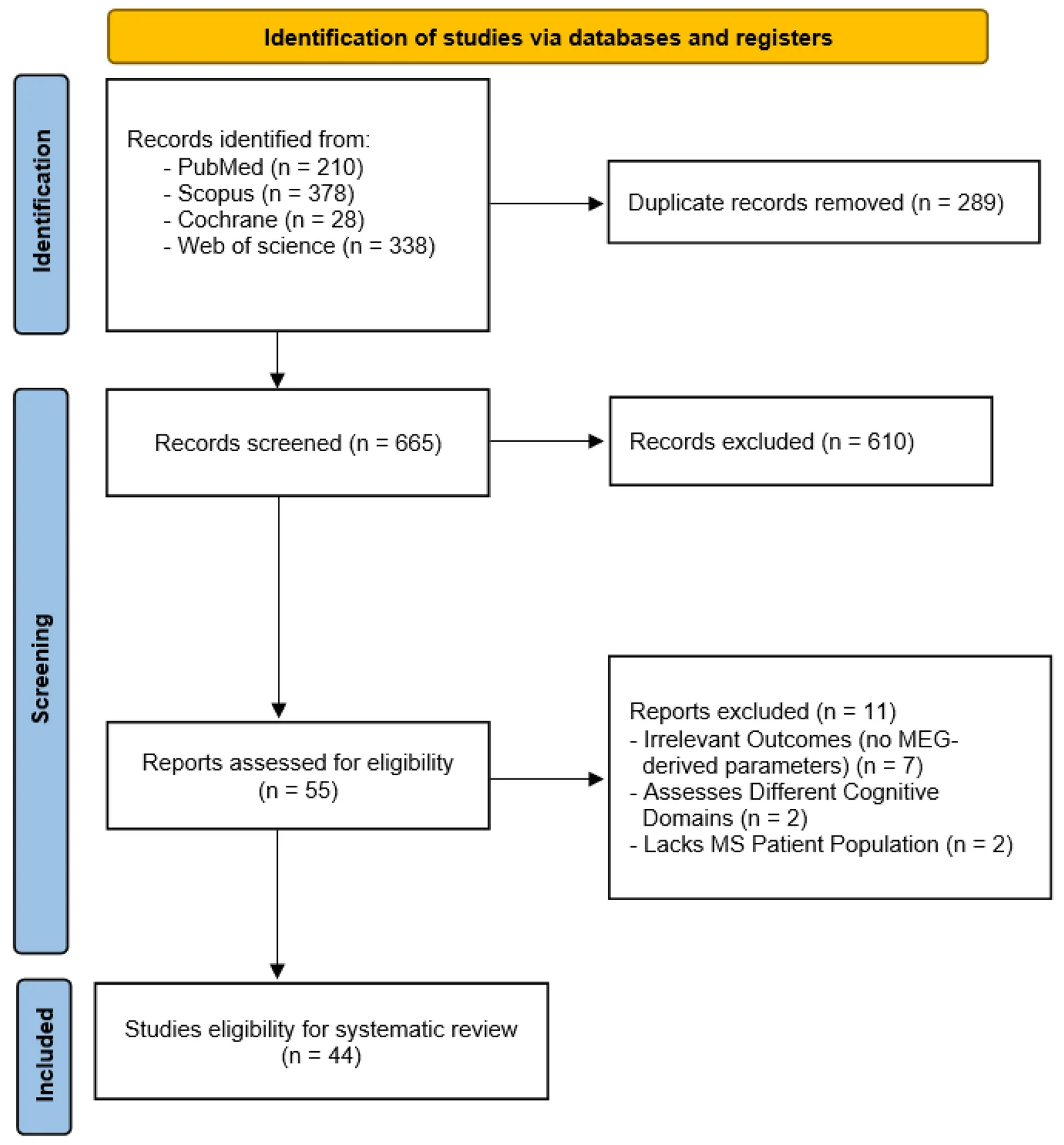
PRISMA Flow diagram.

The initial database search yielded 954 records. Following the removal of 289 duplicates and the screening of 665 titles and abstracts, 55 full-text reports were assessed for eligibility. Eleven reports were excluded at the full-text stage (The 11 reports, along with their specific reasons for exclusion, are listed in Supplementary Table S3). Ultimately, 44 studies [1,3,9,10,11,12,13,14,15,16,32,33,34,35,36,37,38,39,40,41,42,43,44,45,46,47,48,49,50,51,52,53,54,55,56,57,58,59,60,61,62,63,64,65] met the inclusion criteria for this systematic review (Figure 2). The included studies analyzed diverse MS populations, predominantly RRMS. Sample sizes varied significantly, ranging from small pilots (*N* = 10, Kotini 2007) [50] to large-scale cohort analyses (*N* = 209, Bijvank 2021) [54]. The majority of studies compared MS patients to HC, with MS groups frequently exhibiting a female predominance (e.g., 72–80% female in several cohorts). Age demographics generally ranged from 30 to 55 years, except for one pediatric-onset study (*N* = 29, mean age 17.5 ± 3.1). Disability levels, quantified by the EDSS, ranged from minimal disability (EDSS 1.0–2.0) to moderate-severe disability (median EDSS up to 5.5 in Arpin 2017) [35]. Detailed characteristics and results are shown in Table 3. Regarding Quality Assessment, most included studies were rated as “fair” quality, while three studies (Has Silemek 2023 [15]; Nauta 2021, 2024 [13,53]) achieved a “good” rating. The quality assessment for all 44 included studies is provided in Supplementary Table S4.

#### 3.3.2. Resting-State Neural Dynamics: Spectral Power and Slowing

A consistent finding across resting-state studies was the phenomenon of “neuronal slowing,” characterized by a shift in spectral power from higher to lower frequency bands. Multiple studies reported alterations in the alpha band (8–13 Hz). Specifically, MS patients demonstrated a decrease in alpha band power and a shift in the alpha peak toward lower frequencies compared to controls. In cognitively impaired patients (*N* = 117), Schoonhoven et al. (2019) reported increased relative power in the theta (4–8 Hz) and lower alpha (8–10 Hz) bands, alongside a significantly reduced peak frequency (9.2 Hz vs. 9.7 Hz in controls) [10]. Van Der Meer et al. (2013) corroborated this, noting increased alpha1 power and decreased alpha2 power, with the former associated with slower information-processing speed [63]. Regarding broadband dynamics, Akbarian et al. (2023) used aperiodic 1/f slope analysis [33] and found that cognitively impaired MS patients (*N* = 139) exhibited a steeper 1/f slope than preserved patients and controls, suggesting an imbalance in excitation/inhibition. However, a subsequent study by the same group found no significant difference in the pre-stimulus baseline 1/f slope between groups, indicating potential variability based on medication status (e.g., Benzodiazepines) or cohort differences.

#### 3.3.3. Functional Connectivity and Network Topology

Analyses of functional connectivity (FC) and graph theory metrics revealed frequency-dependent network reorganization.

- Alpha Band: Decreased connectivity was a recurrent theme. Sjøgård et al. (2021) reported reduced alpha-band FC within the Default Mode Network (DMN) [12], while Tewarie et al. (2013) observed lower FC in the alpha2 band in visual and DMNs [11].
- Beta Band: Findings in the beta band (13–30 Hz) were mixed. Sjøgård et al. (2021) found reduced FC in the sensorimotor network, which correlated with physical disability [12]. Conversely, Tewarie et al. (2013) and Schoonheim et al. (2013) reported higher beta connectivity or synchronization likelihood in MS patients compared to controls [11,58].
- Network Topology: Graph theory approaches using Minimum Spanning Trees (MST) indicated topological shifts. Tewarie et al. (2014) described a shift toward a “star-like” topology in the theta band (higher leaf fraction) but a more “line-like” topology in the alpha2 band (lower leaf fraction) in early RRMS [14]. Has Silemek et al. (2023) identified a progressive loss of hub connectivity in the DMN and somatomotor networks over a 2-year longitudinal period (*N* = 76) [15].

#### 3.3.4. Event-Related Dynamics and Task Modulation

Studies utilizing active tasks demonstrated significant deviations in neural processing efficiency and inhibition in MS patients.

- Somatosensory Processing: MS patients exhibited impaired somatosensory gating. In paired-pulse stimulation paradigms, healthy controls showed significant attenuation of the secondary response (Peak 2 < Peak 1), whereas MS patients failed to suppress the second stimulus, resulting in a higher gating ratio. This lack of inhibition was correlated with mobility impairments. Additionally, Karhu et al. (1992) reported abnormally large-amplitude deflections at 60–80 ms (P60m) [46].
- Motor Systems (Beta Rebound): A robust finding across multiple studies was the attenuation of the Post-Movement Beta Rebound (PMBR). Arpin et al. (2017), Barratt et al. (2017), and Sanders et al. (2025) consistently reported that MS patients exhibited weaker or delayed PMBR following motor tasks (e.g., knee extension, button press) compared to controls [37,38,57].
- Visual Processing (Gamma Oscillations): Visual stimulation tasks revealed diminished gamma band (30–80 Hz) responses. Reduced gamma amplitude or power has been reported in adult mixed-subtype cohorts (Barratt, 2017; Stickland, 2019) and pediatric-onset MS (*N* = 29, Waldman, 2020) [1,38,65].
- Cognitive Modulation: During auditory oddball and n-back tasks, MS patients showed altered modulation of the 1/f slope, specifically attenuated slope steepening after distractor stimuli, indicating a deficit in dynamic inhibition.

#### 3.3.5. Clinical and Structural Correlations

Physiological abnormalities were frequently linked to clinical outcomes.

- Cognition: Cognitive impairment was strongly associated with spectral slowing (increased theta/alpha1 power), steeper 1/f slopes, and specific topological changes such as increased clustering in the lower alpha band. Lower tree hierarchy in the alpha2 band was also associated with poorer overall cognitive performance.
- Physical Disability: Physical impairment (EDSS) was correlated with reduced beta-band functional connectivity in the sensorimotor network and higher beta DMN connectivity.
- Structure-Function Coupling: Several studies examined the relationship between structural damage and functional metrics. Kulik et al. (2022) found that cognitively impaired patients showed stronger structure-function coupling in the theta band [51]. Furthermore, Mazzara et al. (2025) employed computational modeling to demonstrate that conduction delays caused by lesions were inversely correlated with alpha peak amplitude, indicating that lesion topography, rather than total volume, drives spectral changes [52].

## 4. Discussion

Our study utilized MEG to characterize the dynamic reorganization of functional brain networks in MS patients with high fatigue burden compared with healthy controls. While MS patients exhibited a pattern of neural disconnection during rest (reduced interhemispheric coherence in alpha and beta bands), they demonstrated compensatory hyperconnectivity during the SDMT task. This shift was prominent in the gamma and theta bands, hinting that MS patients recruit additional neural resources to maintain cognitive performance levels comparable to healthy controls. This compensation is further evidenced by the absence of significant differences between MS patients and controls in task accuracy and MEG processing latencies (e.g., VEF, Wernicke, and Cingulate latencies), despite significant neurological disruption and high fatigue levels. Such network hyperconnectivity may alternatively reflect a maladaptive phenomenon driven by diffuse demyelination and a pathological loss of network inhibition, rather than a purely adaptive compensatory response.

The cognitive impact of MS and the elevated fatigue levels often seen in these patients are well documented [18,66], and the association between clinical-cognitive manifestations and lesion load has been detailed previously [67]. Recent resting-state MEG studies have demonstrated its utility for mapping neural activity and altered connectivity in MS; we applied this approach to examine network reorganization in patients with MS with high fatigue burden at rest, identifying compensatory connections related to cognitive processing speed [11,12]. Our resting-state findings, which show reduced connectivity in alpha and beta bands, align with those of Sjøgård et al. [12], who concluded that MS-induced dysconnectivity is a hallmark of the disease. However, our study extends this work by demonstrating that during cognitive demand (SDMT), these networks undergo compensatory reorganization.

The failure of long-range neural communication is likely a direct consequence of white matter degradation, which renders standard physiological pathways less efficient. Conversely, the hyper-synchrony in theta and gamma bands during cognitive effort (SDMT) represents a compensatory functional shift, rather than a simple manifestation of disease. By employing higher-frequency oscillations, the MS brain appears to integrate information through alternative cortical hubs. While this process preserves processing speed and accuracy, as evidenced by our MEG latency and SDMT performance data, it is likely metabolically demanding, which aligns with the significantly elevated cognitive and physical fatigue scores (MFIS) reported in the MS group. This provides a potential explanation for why, despite significant neurological burden and subjective fatigue, MS patients may maintain performance levels indistinguishable from healthy controls in specific cognitive domains. This functional shift is consistent with evidence that frontal gamma oscillations are crucial for modulating working memory [68] and that theta rhythms facilitate long-range communication during memory-intensive tasks [69]. Furthermore, the increased connectivity we observed in the gamma band aligns with findings that link gamma power and distribution to the preservation of cognitive performance [69,70].

Further supporting the compensatory role of gamma oscillations, a long-term cohort study assessed cognitive function in healthy individuals at the ages of 63 and 68 through multiple measures, including a comprehensive neuropsychological battery and electroencephalography with scalp electrodes (measured at steady-state with visual evoked potentials at alpha and gamma frequencies) [70] have shown that while cognitive performance remains stable over five years, the power of gamma oscillations shifts from posterior to anterior brain regions with age. This reorganization is thought to be a neural response to maintaining cognitive stability. Our findings align with this concept, suggesting that MS patients might undergo a process paralleling accelerated cognitive aging. In this context, the disrupted neural pathways in both MS and aging may drive a similar compensatory shift. Thus, the intense interhemispheric gamma-band coherence observed in our patients may be an attempt to recruit alternative networks to bypass structural deficits and maintain performance. This analogy remains speculative, as the aforementioned study examined healthy elderly rather than MS patients, and requires validation in a longitudinal MS cohort.

Our experimental findings provide a critical context for the variability observed in our systematic review of 44 MEG studies. The review confirms both spectral-power neuronal slowing and reduced alpha/beta connectivity at rest [10]; our resting-state coherence findings are parallel to the latter. The task-related dynamics in which our MS cohort demonstrated a significant hyper-synchrony in the gamma and theta bands during the SDMT. Some previous studies, however, reported diminished gamma responses during simple visual stimulation [1,38]. These findings may reflect different neural processes. Passive visual stimulation is thought to drive local, feed forward gamma activity in the visual cortex, which appears attenuated in MS. The SDMT, by contrast, is a high-load cognitive task that may require more distributed information integration, potentially recruiting inter-regional gamma coherence across frontal and parietal networks. If so, reduced local sensory gamma and increased network-level gamma coherence could coexist in the same patient. This interpretation would be consistent with the preserved processing latencies observed in our cohort, although it remains speculative and warrants confirmation in larger studies.

Our study combines MEG analysis with a systematic review of 44 studies, offering a robust framework for interpreting MS-related dysconnectivity. By examining multiple frequency bands during rest and during cognitive challenge (SDMT), we captured compensatory mechanisms in gamma and theta bands that are often missed in resting-state research. Limitations include a small, single-center sample size, limiting statistical power, and a lack of individualized MRI co-registration. Delta band exclusion was necessary to avoid speech artifacts, but future non-verbal paradigms could explore delta dynamics. Longitudinal designs and multimodal imaging are needed to assess compensatory stability and structure–function relationships.

## 5. Conclusions

In summary, our study highlights dynamic neural reorganization in MS patients. While the disease causes a disconnection syndrome at rest (reduced alpha/beta), the brain compensates during cognitive tasks through hyper-synchrony in theta and gamma bands. This neural overdrive offers a potential framework to explain how patients maintain normal processing speed and accuracy despite their underlying neurological burden and high fatigue levels. Supported by a systematic review, these findings position MEG as a potential biomarker for neuroplasticity and provide a foundation for future interventions targeting specific frequency bands to manage cognitive fatigue.

## Figures and Tables

**Table 1:** Baseline characteristics of the included participants.

Variable	MS Subjects (*N* = 10)	Control Subjects (*N* = 7)	*p*-Value ^1^
Age	40.3 ± 9.2	46.7 ± 9.6	0.184
Gender			
Females	9 (90%)	6 (86%)	
Male	1 (10%)	1 (14%)	0.787
SDMT score paper	43.3 ± 11.2	57.7 ± 12.9	**0.027**
SDMT score paper % *	98.7 ± 3.5	99.5 ± 0.9	0.502
SDMT score MEG/110	105.3 ± 5.9	108.0 ± 3.4	0.292
SDMT score MEG/110%	95.6 ± 5.5	98.2 ± 3.1	0.283
MFIS Total score	39.4 ± 18.4	8.4 ± 10.0	**0.001**
MFIS Physical	18.9 ± 9.7	2.1 ± 2.9	**<0.001**
MFIS Cognitive	16.8 ± 7.6	5.4 ± 6.1	**0.005**
MFIS Psycho-social	3.7 ± 2.2	0.9 ± 1.2	**0.007**
MEG-VEF latency	83.0 ± 9.3	81.4 ± 14.0	0.784
MEG-Wernicke latency	220.0 ± 21.7	211.1 ± 30.8	0.495
MEG-Decision cingulate latency	298.4 ± 29.2	305.7 ± 26.3	0.605
MEG-Broca’s frontal latency	381.0 ± 33.6	402.0 ± 22.8	0.172

* SDMT score paper %: calculated as correct responses divided by total items attempted during the 90 s administration, expressed as a percentage. This accuracy metric is distinct from the raw SDMT score (total correct substitutions in 90 s) and captures response precision independent of processing speed. Mean + Standard Deviation; ^1^ *p*-values from the two-sample *t*-test and chi-square test for sex. Bold indicates statistical significance (*p* < 0.05).

**Table 2:** Summary of Functional Connectivity Differences in MEG-Resting and MEG-SDMT states across different frequency bands in Multiple Sclerosis patients and healthy controls.

Frequency Band	Group	MEG-Resting Key Findings	MEG-SDMT Key Findings
**Theta Band (4–7 Hz)** Supplementary Figures S1 and S5	**MS**	Strong coherence across right, left, and interhemispheric regions (L 117, R 131, I 209; total 457)	Increased coherence compared with resting, with overall marked increase vs. HC (L 181, R 145, I 286; total 612), adding the temporal and occipital gyri.
	**Control**	Fewer overall connections (L 60, R 79, I 218; total 357)	Less overall connectivity (L 43, R 66, I 153; total 262)
**Alpha Band (8–13 Hz)** Supplementary Figures S2 and S6	**MS**	Higher coherence in frontal–frontal, frontal–parietal, fusiform–striatal, parietal–striatal pairs; lower interhemispheric (L 92, R 68, I 134; total 294)	More comparable to HC (L 105, R 107, I 202; total 414).
	**Control**	Higher interhemispheric coherence (L 123, R 111, I 298; total 532)	Similar to resting (L 76, R 128, I 272; total 476).
**Beta Band (14–30 Hz)** Supplementary Figures S3 and S7	**MS**	Sparse, highly localized connectivity (L 20, R 0, I 5; total 25)	Higher than at rest, with a left-hemisphere shift (L 67, R 60, I 87; total 214)
	**Control**	Overall higher coherence (interhemispheric, right, left) (L 161, R 209, I 444; total 814).	Lower than at rest but still higher than MS (L 82, R 159, I 312; total 553).
**Gamma Band (30–85 Hz)** Supplementary Figures S4 and S8	**MS**	More localized coherence, fewer overall connections (L 101, R 93, I 171; total 365).	Enhanced connectivity across right, left, and interhemispheric regions (L 183, R 138, I 311; total 632)
	**Control**	Strong coherence in right, left, and interhemispheric regions (L 108, R 127, I 295; total 530)	Less overall connectivity (L 46, R 62, I 157; total 265).

**Table 3:** Findings from the previous studies about MEG Functional Connectivity Studies in Multiple Sclerosis.

Study ID	Country	MS Subtype	N. of Participants	Female, *N* (%)	Age (Years)	EDSS Baseline	Frequency/Metric	Resting State Key Findings (MS vs. Controls)	Task/Active State Key Findings (MS vs. Controls or Modulation from Baseline)
Akbarian 2023 [33]	Belgium	RRMS, SPMS, PPMS, CIS	139 (95 MS, 44 Controls)	MS = 65 (68.4%) HC = 25 (56.8%)	MS = 47.8 ± 9.8 HC = 47 ± 11	(BZD+) = 3.5 [2.75–4.5], (BZD−) = 2.5 [2–3.625]	Broadband (1/f slope)	Cognitively impaired MS patients show a steeper 1/f slope than preserved MS and controls. MS on Benzodiazepines (BZD) shows a steeper slope than MS without BZD.	N/A (Resting state study)
Akbarian 2024 [34]	Belgium	MS (diagnosed via McDonald criteria)	117 (79 MS, 38 Controls)	MS = 57 (72.2%) HC = 23 (60.5%)	MS = 48 ± 9.9 HC = 47 ± 11	(BZD+) = 3.5 [3–4.25], (BZD−) = 2.5 [2–3.5]	Broadband (1/f slope)	No significant difference in pre-stimulus (baseline) 1/f slope between MS and controls	Post-Distractor Stimulus: MS patients showed a significantly flatter 1/f slope (less inhibition) compared to controls. General Modulation: Both groups showed 1/f steepening (increased inhibition) after target stimuli.
Akbarian 2025 [32]	Belgium	MS (diagnosed via McDonald criteria)	126 (82 MS, 44 Controls)	MS = 60 (73.2%) HC = 26 (59.1%)	MS = 47.8 ± 9.7 HC = 47 ± 12	(BZD+) = 3 [2.5–4], (BZD−) = 2.5 [2–4]	Broadband (1/f slope)	N/A (Focus on task modulation)	Auditory Oddball: MS patients (on BZD) showed attenuated slope steepening (less inhibition) after distractor stimuli compared to controls. Cross-Task: 1/f slope modulation was correlated between the auditory oddball and n-back tasks.
Arpin 2017 (1) [35]	USA	RRMS, SPMS	23 (11 MS, 12 Controls)	MS = 9 (81.8%) HC = 9 (75%)	MS = 56.1 ± 6 HC = 54.7 ± 7	5.5 ± 0.8	Somatosensory Evoked Fields (SEF) (Metric: Gating Ratio & Peak Amplitude)	N/A (Study design focused on event-related responses to stimulation)	Somatosensory Gating (Paired-Pulse Stimulation): MS patients exhibited reduced somatosensory gating compared to healthy controls. While controls showed significant attenuation of the neural response to the second stimulus (Peak 2 < Peak 1), MS patients did not show this suppression, resulting in a significantly higher gating ratio. Response Amplitude: The amplitude of the second response (Peak 2) was significantly larger in MS patients than in controls, while the first response (Peak 1) did not differ between groups. Behavioral Correlation: Weaker somatosensory gating (higher ratio) was significantly correlated with mobility impairments, specifically slower walking velocity and shorter step length.
Arpin 2017 (2) [37]	USA	RRMS, SPMS	30 (15 MS, 15 Controls)	MS = 12 (80%) HC = 12 (80%)	MS = 56.1 ± 6.5 HC = 55.1 ± 6.9	5.5 ± 0.7	Beta (15–30 Hz)	N/A (Focus on motor task)	Knee Extension: MS patients exhibited a significantly weaker Post-Movement Beta Rebound (PMBR) compared to controls. No difference in beta ERD (desynchronization) during movement planning.
Arpin 2018 [36]	USA	RRMS, SPMS	30 (15 MS, 15 Controls)	MS = 12 (80%) HC = 12 (80%)	MS = 56.1 ± 6.5 HC = 55.1 ± 6.9	5.5 ± 0.7	Somatosensory Gating	N/A (Focus on gating)	Paired-Pulse Stimulation: Controls showed significant attenuation (gating) of the second stimulus response; MS patients did not show significant gating (amplitude of second response was larger in MS than controls)
Barratt 2017 [38]	UK	RRMS, PPMS, SPMS	43 (21 MS, 22 Controls)	MS = 12 (57.1%) HC = 12 (54.5%)	MS = 42 ± 11 HC = 42 ± 12	3 [0–6]	Beta (13–30 Hz) & Gamma (30–70 Hz)	No significant difference in resting state motor beta or visual gamma amplitude	Visuomotor Task: MS patients showed a significant delay (increased time-to-peak) of the Post-Movement Beta Rebound (PMBR). MS patients showed significantly reduced visual gamma oscillation amplitude.
Costers 2021 [41]	Belgium	RRMS, PPMS, SPMS, CIS	117 (79 MS, 38 Controls)	MS = 57 (72.2%) HC = 23 (60.5%)	MS = 47.9 ± 9.6 HC = 48.0 ± 12.0	3 [2–4]	Theta (4–8 Hz)	N/A (Focus on N-back)	N-back Task: MS patients showed a smaller maximum theta-power increase in the right hippocampus (0–400 ms post-stimulus) than controls.
Cover 2006 [9]	The Netherlands	RRMS	21 (10 MS, 11 Controls)	MS = 9 (90%) HC = 5 (45.5%)	MS = 40.5 [25–55] HC = 29.2 [23–48]	[1–3.5]	Alpha (8–12 Hz)	MS patients show a highly significant decrease in interhemispheric coherence.	N/A (Resting state study)
Cipriano 2023 [40]	Italy	RRMS, SPMS	50 (25 MS, 25 Controls)	MS = 18 (72%) HC = 18 (72%)	MS = 45.7 ± 9.5 HC = 45.8 ± 11.8	4.5 [1.5–7.0]	Alpha (Metric: Clinical Connectome Fingerprinting via PLM)	MS patients showed significantly lower identifiability scores (I-self, I-others, and I-diff) in the alpha band compared to healthy controls. This implies a lower similarity between two functional connectomes (FCs) of the same patient and reduced homogeneity among FCs in the MS group. Furthermore, reduced identifiability (specifically the I-clinical score) in the alpha band significantly predicted individual fatigue levels (FSS).	N/A (Resting state study)
Cipriano 2024 [39]	Italy	RRMS, SPMS	50 (25 MS, 25 Controls)	MS = 18 (72%) HC = 18 (72%)	MS = 45.7 ± 9.5 HC = 45.8 ± 11.8	RRMS = 3.8 [1.5–6.0] SPMS = 5.1 [2.5–7.0]	Broadband (0.5–48 Hz) (Metric: Functional Repertoire)	MS patients showed a significantly larger functional repertoire (a greater number of unique brain reconfigurations) compared to healthy controls. However, this difference in brain flexibility was primarily driven by patients with the RRMS phenotype; dynamics in SPMS patients did not differ significantly from healthy controls. Additionally, brain flexibility showed different predictive powers depending on the MS type: in RRMS subjects, a larger functional repertoire was significantly associated with a worse clinical condition (positively correlating with EDSS and BDI depression scores and negatively with SDMT cognitive scores).	N/A (Resting state study)
Dell’Acqua 2010 [42]	Italy	RRMS	42 (21 MS, 21 Controls)	MS = 16 (76%) HC = 16 (76%)	MS = 39 ± 9 HC = 38 ± 12	1.5 [0–3.5]	Broadband (SEF) (Metric: Morphology & Similarity)	N/A (Study focuses on evoked responses to median nerve stimulation)	Somatosensory Stimulation: MS patients showed preserved primary sensory cortex (S1) activation (M20 component) but highly distorted morphology of the subsequent response (M30 component). This resulted in a reduced inter-hemispheric similarity index (Morf_S1M1) compared to controls.
Georgopoulos 2007 [43]	USA	RRMS, SPMS	142 (12 MS, 130 others/controls)	MS = 8 (66.7%)	MS = 40.7 ± 3.3	NR	Broadband (1–400 Hz) (Metric: Synchronous Neural Interactions)	Fixation (Rest): Pairwise zero-lag partial cross-correlations (PCC) successfully classified MS patients from healthy controls (and other disease groups) with 100% accuracy using specific predictor subsets of sensor pairs	N/A
Has Silemek 2023 [15]	Germany	RRMS	76 (37 MS, 39 Controls)	MS = 22 (59.5%) HC = 22 (56.4%)	MS = 42.2 ± 9.5 HC = 42.2 ± 8.3	2 [1,2]	Alpha (8–13 Hz) (Connectivity: Multivariate Interaction Measure)	Hub Disruption: MS patients showed significantly lower hub connectivity (degree) in the Default Mode, Somatomotor, and Frontoparietal networks at baseline, compared to controls. Longitudinal Change: Over 2 years, MS patients exhibited a progressive loss of hub connectivity in the DMN, Somatomotor, and Frontoparietal networks, and dynamic decreases in Dorsal/Ventral Attention networks. Cognition: No significant association was observed between resting-state MEG network metrics and offline cognitive performance in MS.	N/A (Resting State Study with offline neuropsychological testing)
Hagiwara 2010 [44]	Japan	RRMS, SPMS, PPMS	46 (23 MS, 23 Controls)	MS = 18 (78.3%) HC = 18 (78.3%)	MS = 38.8 ± 8.1 HC = 37.3 ± 10.6	2.7 ± 2.7	Gamma (30–70 Hz)	N/A (Focus on sensory task)	Sensory Stimulation: MS patients showed significantly decreased Phase Locking Values (PLV) (synchrony) between primary (SI) and secondary (SII) somatosensory cortices compared to controls
Hardmeier 2012 [45]	The Netherlands	RRMS	62 (34 MS, 28 Controls)	MS = 17 (50%) HC = 14 (50%)	MS = 41.4 ± 8.0 HC = 39.8 ± 10.5	2 [0–4.5]	Theta (4–8 Hz) & Beta (13–30 Hz) (Metric: Eigenvector Centrality)	Theta: MS patients showed higher centrality in bi-parietal hub regions compared to controls. Beta/Upper Alpha: MS patients showed lower centrality in left temporal regions. Lower temporal centrality correlated with cognitive dysfunction.	N/A
Karhu 1992 [46]	Finland	Clinically Definite MS	18 (10 MS, 8 Controls)	MS = 2 (20%) HC = 2 (25%)	MS = 34 [22–56] HC = 32 [22–49]	NR	Evoked Response	N/A (Focus on sensory task)	Sensory Stimulation: MS patients showed abnormally large-amplitude deflections at 60–80 ms (P60m) compared to controls. Early deflections (N20m) were often delayed or suppressed.
Kassubek 1999 [47]	Germany	Definite Laboratory-Supported MS	16 (8 MS, 8 Controls)	MS = 4 (50%) HC = 1 (12.5%)	MS = 32 [18–49] HC = 30 [26–37]	2.5	Slow (2–6 Hz) & Beta (14–30 Hz) (Metric: Dipole Density)	Focal Activity: MS patients exhibited focal abnormal magnetic activity (both slow and beta waves) concentrated in cortical areas adjacent to subcortical lesions. The standardized maximum dipole density was significantly higher in MS patients than in controls.	N/A
Kim 2019 [48]	Canada	RRMS, RIS	53 (27 MS, 26 Controls)	MS = 17 (63%) HC = 16 (61.5%)	MS = 39 ± 10 HC = 35 ± 9	2 ± 2	Alpha (8–13 Hz)	MS patients showed increased power in the ascending nociceptive pathway (thalamus, posterior insula) and salience network (TPJ)	N/A (Resting State Study)
							Beta (13–30 Hz)	MS patients showed decreased power in the thalamus, posterior insula, and salience network.	N/A (Resting State Study)
Kim 2020 [49]	Canada	RRMS, SPMS, RIS	63 (33 MS, 30 Controls)	MS = 21 (63.6%) HC = 19 (63.3%)	MS = 39 ± 10 HC = 35 ± 9	2 ± 2	Theta, Alpha, Beta, Gamma (Metric: Static and Dynamic Functional Coupling)	Cross-Network: MS chronic pain patients showed abnormal cross-network coupling (e.g., between Salience and Ascending Nociceptive networks). Dynamics: Dynamic functional coupling was attenuated in theta and alpha bands in MS patients. Neuropathic Pain: Specific abnormalities found in the neuropathic pain subgroup (e.g., reduced static coupling in alpha/beta bands).	N/A
Kotini 2007 [50]	Greece	Definite MS	10 (5 MS, 5 Controls)	NR	NR	NR	Broadband (Metric: Nonlinear Analysis/Chaos)	Complexity: MS patients showed lower Correlation Dimension (D) values in temporal regions compared to controls, indicating reduced chaotic activity and lower complexity in the MS brain	N/A
Kulik 2022 [51]	The Netherlands	RRMS, SPMS, PPMS	119 (79 MS, 40 Controls)	MS = 57 (72%) HC = 25 (63%)	MS = 53.8 ± 10.7 HC = 50.7 ± 6.11	3.5 [1–8]	Theta (4–8 Hz) (Metric: Structure-Function Coupling)	Coupling Strength: Cognitively impaired (CI) MS patients showed stronger long-range structure-function coupling (higher correlation between structural and functional connectivity) compared to healthy controls. SC-FC Relationship: Whole-brain structural connectivity was significantly related to whole-brain functional connectivity in the theta band in MS patients, but not in healthy controls. Biomarker: Structure-function coupling was not a significant classifier of cognitive impairment (low AUC).	N/A (Resting state study with Offline Neuropsychological Testing)
Mazzara 2025 [52]	Italy	MS (diagnosed via McDonald criteria)	38 (18 MS, 20 Controls)	MS = 12 (66.7%) HC = 14 (70%)	MS = 44.9 ± 9.9 HC = 45.8 ± 11	4.5 ± 1.9	Alpha (8–13 Hz) (Metric: Spectral Power & Computational Model Parameter)	Spectral Power: MS patients exhibited a decrease in alpha band power and a shift in the alpha frequency peak towards lower frequencies compared to healthy controls. Lesion Modeling: The study defined a parameter representing the impact of structural lesions on conduction delays; the inferred values were inversely correlated with the amplitude of the alpha peak (higher linked to reduced alpha). Structure-Function: Empirical alpha power peaks did not correlate with total lesion volume, suggesting the specific topography of lesions drives spectral changes rather than total volume load.	N/A (Study design involved MEG recordings during an eyes-closed resting-state condition only)
Nauta 2021 [13]	The Netherlands	RRMS, SPMS, PPMS	206 (146 MS, 60 Controls)	MS = 98 (67.1%)	MS = 48.3 ± 11.2	3 [0–8]	Delta (0.5–4 Hz) & Beta (13–30 Hz) (Metric: Minimum Spanning Tree)	Cognitive Decline: A less integrated delta network (lower leaf fraction) and a more integrated beta network (smaller diameter) at baseline predicted cognitive decline after 5 years. General: Lower tree hierarchy (less integration) is related to worse cognition cross-sectionally.	N/A
Nauta 2024 [53]	The Netherlands	RRMS, SPMS, PPMS, CIS	161 (105 MS, 56 Controls)	MS = 79 (75%) HC = 34 (61%)	MS = 48.6 ± 10 HC = 47.8 ± 9.7	MS Cognitive Preserved = 3.5 [2–6], MS Cog. Impaired = 4 [2–7.5]	Alpha/Theta/Beta	CI MS patients had higher theta and beta PLI (connectivity) and lower gamma power than CP MS patients.	N/A (Study focuses on predicting treatment response from baseline)
Bijvank 2021 [54]	The Netherlands	RRMS, SPMS, PPMS	209 (176 MS, 33 Controls)	MS = 121 (69%) HC = 21 (64%)	MS = 54 ± 10.8 HC = 48.5 ± 9.3	3.5 [2.5–8]	Beta/Theta	Higher pro-saccade latency (eye movement impairment) was associated with lower Beta FC (precuneus) and higher Theta FC.	N/A (Correlated resting MEG with offline eye movement behavior)
Paolicelli 2021 [55]	Italy	RRMS, CIS	35 (16 MS, 19 Controls)	MS = 14 (87.5%) HC = 13 (68.4%)	MS = 36.5 ± 7.5 HC = 36.0 ± 16.0	2 ± 1	ERP/ERF (P300)	N/A (Focus on Oddball)	Acoustic Oddball: MS patients showed similar P300 amplitude and latency to controls (early stage MS). However, time-frequency analysis showed less positive/more negative modulation in alpha/beta in patients.
Rossi 2024 [56]	Belgium	RRMS	108 (70 MS, 38 Controls)	MS = 52 (74.3%) HC = 23 (60.5%)	MS = 47 ± 11.5 HC = 47.5 ± 9.5	[2.5–3.5]	Broadband (Theta & Beta) (Metric: ER Activation Profile via TDE-HMM)	N/A (This was a task-based study).	The study utilized a visual-verbal n-back working memory task. MS patients exhibited significantly decreased activation of an early theta prefrontal network (State 1), which is linked to stimulus encoding and attentional control, compared to healthy controls. This decreased activation significantly correlated with reduced response accuracy and higher reaction times. A frontoparietal network characterized by beta coupling (State 5), which activates 300 to 600 ms post-stimulus (resembling the traditional EEG P300, termed here as “M300”), was significantly amplified in MS patients treated with benzodiazepines.
Sanders 2025 [57]	UK	RRMS, SPMS, PPMS	40 (20 MS, 20 Controls)	MS = 14 (70%) HC = 15 (75%)	MS = 49.7 [35.7–55.8] HC = 46.3 [33.9–54.3]	2 [1.6–3.4]	Beta (13–30 Hz)	Standing vs. Sitting: Standing decreased beta connectivity in the sensorimotor network (significant in controls) and beta power (significant in MS)	Visuomotor Task: MS patients showed a slower rise in the post-movement beta rebound (PMBR) gradient compared to controls.
							Gamma (35–70 Hz)	N/A	Visual Stimulation: MS patients showed diminished gamma amplitude response to visual stimulation compared to controls.
Schoonheim 2013 [58]	The Netherlands	RRMS	62 (34 MS, 28 Controls)	MS = 17 (50%) HC = 14 (50%)	MS = 41.4 ± 8 HC = 39.8 ± 10.5	2 [0–4.5]	Theta, Lower Alpha (8–10 Hz), Upper Alpha (10–13 Hz), Beta (Metric: Synchronization Likelihood-SL & Graph Topology)	Connectivity: MS patients showed higher functional connectivity (SL) in the theta, lower alpha, and beta bands, but lower connectivity in the upper alpha band compared to controls. Topology: In the lower alpha band, MS patients exhibited a shift toward a more regular network (increased path length and clustering coefficient. Clinical: Increased clustering in the lower alpha band was the strongest predictor of cognitive impairment (specifically, attention and working memory). Male patients were more severely affected than females.	N/A (Study design involved eyes-closed resting-state recordings only)
Schoonhoven 2019 [10]	The Netherlands	RRMS, SPMS, PPMS	117 (83 MS, 34 Controls)	MS Cognitive Preserved = 27 (73%) MS Mild Cog. Imp. = 11 (61%) MS Cog. Impaired = 17 (61%) HC = 21 (62%)	MS Cognitive Preserved = 51.9 ± 9.9 MS Mild Cog. Imp. = 54.6 ± 8.6 MS Cog. Impaired = 53.9 ± 9.9 HC = 50.7 ± 9.8	MS Cognitive Preserved = 3.0 (IQR 2.3), MS Mild Cog. Imp. = 3.25 (IQR 2.3), MS Cog. Impaired = 4.5 (IQR 2.5)	Alpha1 (8–10 Hz) & Theta (4–8 Hz) (Metric: Relative Power)	Slowing: Cognitively impaired MS patients showed increased relative power in theta and alpha1 bands (neuronal slowing) compared to controls, particularly in parietotemporal areas. Peak Frequency: Cognitively impaired patients had a significantly lower peak frequency (9.2 Hz) compared to controls (9.7 Hz).	N/A
Simon 2023 [3]	The Netherlands	RRMS, SPMS, PPMS, CIS	90 (90 MS)	MS = 60 (66.7%)	MS = 47.3 ± 9.6	4 (IQR 1.0)	Broadband (Delta, Theta, Alpha1, Alpha2, Beta & Gamma) (Metric: Relative Power, Peak Frequency, PLI & AECc)	Cognitively Impaired patients exhibited “neuronal slowing,” showing higher relative power in low-frequency bands (theta and alpha1) and lower relative power in high-frequency bands (alpha2 and beta) compared to Cognitively Preserved patients. CI patients also demonstrated lower resting-state functional connectivity (specifically lower delta and gamma PLI) compared to the CP group. Notably, the suboptimally performing (SUB) patients had functional profiles that did not differ significantly from those of CP patients, suggesting their neurophysiology resembled that of cognitively preserved individuals more than that of impaired individuals.	N/A
Sjøgård 2021 [12]	Belgium	RRMS, Progressive MS	146 (99 MS, 47 Controls)	MS = 68 (68.7%) HC = 29 (61.7%)	MS = 47.7 ± 9.7 HC = 47.8 ± 11.9	3 [1–6.5]	Beta (13–30 Hz) & Alpha (8–12 Hz) (Metric: Power Envelope Correlation)	Sensorimotor: MS patients showed reduced functional connectivity (FC) in the sensorimotor network (beta band), which correlated with physical disability. DMN: Reduced alpha-band FC within the Default Mode Network and between DMN and other networks.	N/A
Sorrentino 2024 [59]	Italy	MS (diagnosed via McDonald criteria)	38 (18 MS, 20 Controls)	MS = 12 (66.7%) HC = 14 (70.0%)	MS = 44.9 ± 9.9 HC = 45.8 ± 11.0	4.5 ± 1.9	Alpha (8–13 Hz) (Metric: Power Spectral Density & Conduction Velocities)	MS patients exhibited significantly attenuated peak alpha amplitudes and lower total power in the alpha band compared to controls. Through model inversion, the study demonstrated a mechanistic link between these spectral changes and demyelination, finding that the inferred whole-brain conduction velocities were significantly slower in the MS group compared to controls. Furthermore, these inferred individualized conduction velocities were superior predictors of clinical disability (EDSS) than structural damage measures such as total lesion load or global atrophy.	N/A
Stickland 2019 [1]	UK	MS (diagnosed via McDonald criteria)	23 (13 MS, 10 Controls)	MS = 9 (64.3%) HC = 9 (90%)	MS = 43.46 ± 3.5 HC = 42.4 ± 3.73	3.0 [0–4.5]	Gamma (30–80 Hz)	No significant group differences in baseline gamma power.	Visual Stimulation: MS patients showed a significant reduction in peak gamma power change (and BOLD/CBF response) compared to controls
Tewarie 2017 [60]	The Netherlands	RRMS, SPMS, PPMS	102 (102 MS)	MS = 66 (64.7%)	MS = 54.3 ± 10	4.1 [1–8]	Alpha (8–13 Hz) & Delta (0.5–4 Hz) (Metric: Power & PLI)	Structure-Function: In MS with optic neuritis (MSON), alpha band connectivity was positively related to outer retinal layer thickness. In MS without optic neuritis (MSNON), connectivity was related to retinal thickness in the delta band.	N/A
Tewarie 2013 [11]	The Netherlands	RRMS	38 (21 MS, 17 Controls)	MS = 12 (57%) HC = 10 (59%)	MS = 41.9 ± 7.7 HC = 39.8 ± 9.8	2 [0–4.5]	Alpha2 (10–13 Hz) & Beta (13–30 Hz) (Metric: Phase Lag Index)	Connectivity: MS patients showed lower FC in the alpha2 band (visual and DMNs) and higher FC in the beta band (DMN and temporo-parietal networks). Clinical: Higher beta DMN connectivity correlated with higher disability (EDSS) and poorer cognition.	N/A
Tewarie 2014 (1) [62]	The Netherlands	RRMS, SPMS, PPMS	144 (102 MS, 42 Controls)	MS = 65 (63.7%) HC = 26 (61.9%)	MS = 54.23 ± 9.76 HC = 51.14 ± 5.98	2.5 [0–6.5]	Alpha2 (10–13 Hz)	MS patients showed lower leaf fraction and tree hierarchy (more random topology)	N/A (Resting state study)
							Theta (4–7 Hz)	MS patients showed higher leaf fraction and lower diameter (more regular/integrated topology)	
Tewarie 2015 [61]	The Netherlands	RRMS, SPMS, PPMS	107 (86 MS, 21 Controls)	MS = 12 (57%) HC = 10 (59%)	MS = 41.9 ± 7.7 HC = 39.8 ± 9.8	2 [0–4.5]	Theta (4–8 Hz) (Metric: Minimum Spanning Tree)	Topology: MS patients showed lower integration (lower leaf fraction/degree divergence) in the theta band. This topology was associated with thalamic atrophy and increased BOLD thalamocortical connectivity.	N/A
Tewarie 2014 (2) [14]	The Netherlands	RRMS	38 (21 MS, 17 Controls)	MS = 12 (57%) HC = 10 (59%)	MS = 41.9 ± 7.7 HC = 39.8 ± 9.8	2 [0–4.5]	Theta (4–8 Hz) (Metric: Minimum Spanning Tree-MST)	Topology: Early RRMS patients showed a shift toward a more star-like topology, characterized by a higher leaf fraction, lower diameter, and lower eccentricity compared to healthy controls.	N/A
							Alpha2 (10–13 Hz) (Metric: Minimum Spanning Tree-MST)	Topology: Early RRMS patients showed a shift toward a more path-like (line-like) topology, characterized by a lower leaf fraction, lower tree hierarchy, lower kappa, and higher diameter/eccentricity. Cognition: Lower tree hierarchy in the alpha2 band was significantly associated with poorer overall cognitive performance in MS patients.	
							Beta (13–30 Hz) (Metric: Minimum Spanning Tree-MST)	Topology: Early RRMS patients showed significantly higher mean eccentricity compared to controls, mainly in frontal and parietal areas.	
Van Der Meer 2013 [63]	The Netherlands	RRMS	38 (21 MS, 17 Controls)	MS = 12 (57%) HC = 10 (59%)	MS = 41.9 ± 7.7 HC = 39.8 ± 9.8	2 [0–4.5]	Alpha1 (8–10 Hz) & Alpha2 (10–13 Hz) (Metric: Relative Power)	Slowing: MS patients showed increased power in the alpha1 band and decreased power in the alpha2 band. Cognition: Increased alpha1 power was associated with worse overall cognition and information processing speed.	N/A
Van Schependom 2019 [64]	Belgium	RRMS, Progressive MS	136 (90 MS, 46 Controls)	MS = 63 (70%) HC = 27 (58.7%)	MS = 48.2 ± 9.5 HC = 47.8 ± 12	(BZD+) = 3.25 [2.5–4.5], (BZD−) = 2.5 [2–4],	Broadband (HMM States)	Dynamics: Male MS patients showed a less dynamic frontal DMN (longer lifetimes), while female patients showed reduced activation of this network. Medication: Benzodiazepines increased beta power and time spent in the deactivating sensorimotor network.	N/A
Van Schependom 2021 [16]	Belgium	RRMS	114 (67 MS, 47 Controls)	MS = 38 (57.6%) HC = 28 (60.9%)	MS = 49 ± 10 HC = 47 ± 12	2.5 [2,3]	Lower Alpha (8–9 Hz) (Metric: Power Spectral Density)	Atrophy: Increased whole-brain atrophy and lesion load were associated with increased lower alpha power in the temporoparietal junction (TPJ). Cognition: Higher TPJ, lower alpha power correlated with worse verbal and spatial working memory.	N/A
Waldman 2020 [65]	USA	Pediatric-onset RRMS	29 (14 MS, 15 Controls)	MS = 6 (42.9%) HC = 10 (66.7%)	MS = 17.5 ± 3.1 HC = 18.7 ± 2	1 (0–3.5)	Visual Gamma (30–80 Hz)	N/A	Visual Stimulation: Pediatric MS (POMS) showed reduced visual gamma power compared to controls
							Motor Beta (15–30 Hz)		Button Press: POMS showed decreased Post-Movement Beta Rebound (PMBR) amplitude compared to controls

## Data Availability

The experimental MEG datasets analyzed in the current study are not publicly available due to patient privacy and ethical restrictions but are available from the corresponding author upon reasonable request. The data supporting the systematic review findings are available within the article and its Supplementary Materials.

## Supplementary Materials

The following supporting information can be downloaded at: https://www.mdpi.com/article/10.3390/brainsci16080862/s1. Supplementary Table S1: Full search strategies for each database, including all controlled vocabulary and free-text terms. Supplementary Table S2. Completed PRISMA 2020 checklist indicating the location of each reporting item within the manuscript. Supplementary Table S3: Full-text reports excluded from the systematic review (*n* = 11), with the specific reason for exclusion for each study. Supplementary Table S4: Risk-of-bias and quality assessment for all 44 included studies, rated using the NIH quality assessment tool. Supplementary Figure S1: Resting-state theta band connectivity differences between MS patients and HCs. (A) Spatial distribution of theta band connections on MRI; green indicates higher coherence in HC, red in MS; only differences between groups are shown. (B) Hemispheric connectivity counts comparison between groups. (C) Adjacency matrix of region-to-region connectivity in MS patients. (D) Adjacency matrix of region-to-region connectivity in healthy controls; Supplementary Figure S2: Resting-state alpha band connectivity differences between MS patients and HC. (A) Spatial distribution of alpha band connections on MRI; green indicates higher coherence in HC, red in MS; only differences between groups are shown. (B) Hemispheric connectivity counts comparison between groups. (C) Adjacency matrix of region-to-region connectivity in MS patients. (D) Adjacency matrix of region-to-region connectivity in healthy controls; Supplementary Figure S3: Resting-state beta band connectivity differences between MS patients and HC. (A) Spatial distribution of beta band connections on MRI; green indicates higher coherence in HC, red in MS; only differences between groups are shown. (B) Hemispheric connectivity counts comparison between groups. (C) Adjacency matrix of region-to-region connectivity in MS patients. (D) Adjacency matrix of region-to-region connectivity in healthy controls; Supplementary Figure S4: Resting-state gamma band connectivity differences between MS patients and healthy controls. (A) Spatial distribution of gamma band connections on MRI; green indicates higher coherence in HC, red in MS; only differences between groups are shown. (B) Hemispheric connectivity counts comparison between groups. (C) Adjacency matrix of region-to-region connectivity in MS patients. (D) Adjacency matrix of region-to-region connectivity in healthy controls; Supplementary Figure S5: Theta band connectivity differences during SDMT performance between MS patients and healthy controls. (A) Task-related theta band connectivity displayed on MRI; green indicates higher coherence in HC, red in MS; only differences between groups are shown. (B) Hemispheric connectivity counts during SDMT. (C) Adjacency matrix of region-to-region connectivity in MS patients during task performance. (D) Adjacency matrix of region-to-region connectivity in healthy controls during task performance; Supplementary Figure S6: Alpha band connectivity differences during SDMT performance between MS patients and healthy controls. (A) Task-related alpha band connectivity displayed on MRI; green indicates higher coherence in HC, red in MS; only differences between groups are shown. (B) Hemispheric connectivity counts during SDMT. (C) Adjacency matrix of region-to-region connectivity in MS patients during task performance. (D) Adjacency matrix of region-to-region connectivity in healthy controls during task performance; Supplementary Figure S7: Beta band connectivity differences during SDMT performance between MS patients and healthy controls. (A) Task-related beta band connectivity displayed on MRI; green indicates higher coherence in HC, red in MS; only differences between groups are shown. (B) Hemispheric connectivity counts during SDMT. (C) Adjacency matrix of region-to-region connectivity in MS patients during task performance. (D) Adjacency matrix of region-to-region connectivity in healthy controls during task performance; Supplementary Figure S8: Gamma band connectivity differences during SDMT performance between MS patients and healthy controls. (A) Task-related gamma band connectivity displayed on MRI; green indicates higher coherence in HC, red in MS; only differences between groups are shown. (B) Hemispheric connectivity counts during SDMT. (C) Adjacency matrix of region-to-region connectivity in MS patients during task performance. (D) Adjacency matrix of region-to-region connectivity in healthy controls during task performance.

## Abbreviations

The following abbreviations are used in this manuscript:

BDI	Beck Depression Inventory
BZD	Benzodiazepine
CC BY	Creative Commons Attribution
CIS	Clinically Isolated Syndrome
CMSC	Consortium of Multiple Sclerosis Centers
DMN	Default Mode Network
DMT	Disease-Modifying Therapy
EDSS	Expanded Disability Status Scale
ERD	Event-Related Desynchronization
ERF	Event-Related Field
FC	Functional Connectivity
FDR	False Discovery Rate
fMRI	Functional Magnetic Resonance Imaging
FSS	Fatigue Severity Scale
HC	Healthy Control
ICA	Independent Component Analysis
IRB	Institutional Review Board
MAGNIMS	Magnetic Resonance Imaging in Multiple Sclerosis
MEG	Magnetoencephalography
MFIS	Modified Fatigue Impact Scale
MHI	Mental Health Inventory
MRI	Magnetic Resonance Imaging
MS	Multiple Sclerosis
NAIMS	North American Imaging in Multiple Sclerosis
NIH	National Institutes of Health
N-back	N-back Working Memory Task
PCC	Partial Cross-Correlation
PET	Positron Emission Tomography
PLI	Phase Lag Index
PLM	Phase Linearity Measurement
PLV	Phase Locking Value
PMBR	Post-Movement Beta Rebound
PPMS	Primary Progressive Multiple Sclerosis
PRISMA	Preferred Reporting Items for Systematic Reviews and Meta-Analyses
RIS	Radiologically Isolated Syndrome
ROI	Region of Interest
RRMS	Relapsing-Remitting Multiple Sclerosis
SD	Standard Deviation
SDMT	Symbol Digit Modalities Test
SEF	Somatosensory Evoked Field
SI	Primary Somatosensory Cortex
SII	Secondary Somatosensory Cortex
SL	Synchronization Likelihood
SPMS	Secondary Progressive Multiple Sclerosis
TDE-HMM	Time-Delay Embedded Hidden Markov Model
TPJ	Temporoparietal Junction
VAMC	Veterans Affairs Medical Center
VEF	Visual Evoked Field
VHA	Veterans Health Administration
