# Supplementary material for: Functional Connectivity at Rest and During Cognitive Processing in Multiple Sclerosis: A Pilot Magnetoencephalography Study and Systematic Review
Source: Brain Sci. 2026 Aug 14;16(8):862. doi: 10.3390/brainsci16080862 (PMC13511113; doi:10.3390/brainsci16080862)
Supplement: Supplementary file 1 [file brainsci-16-00862-s001.zip › Supplementary figures/Supplementary Figure S1, Theta band - resting state.pdf]

(A)

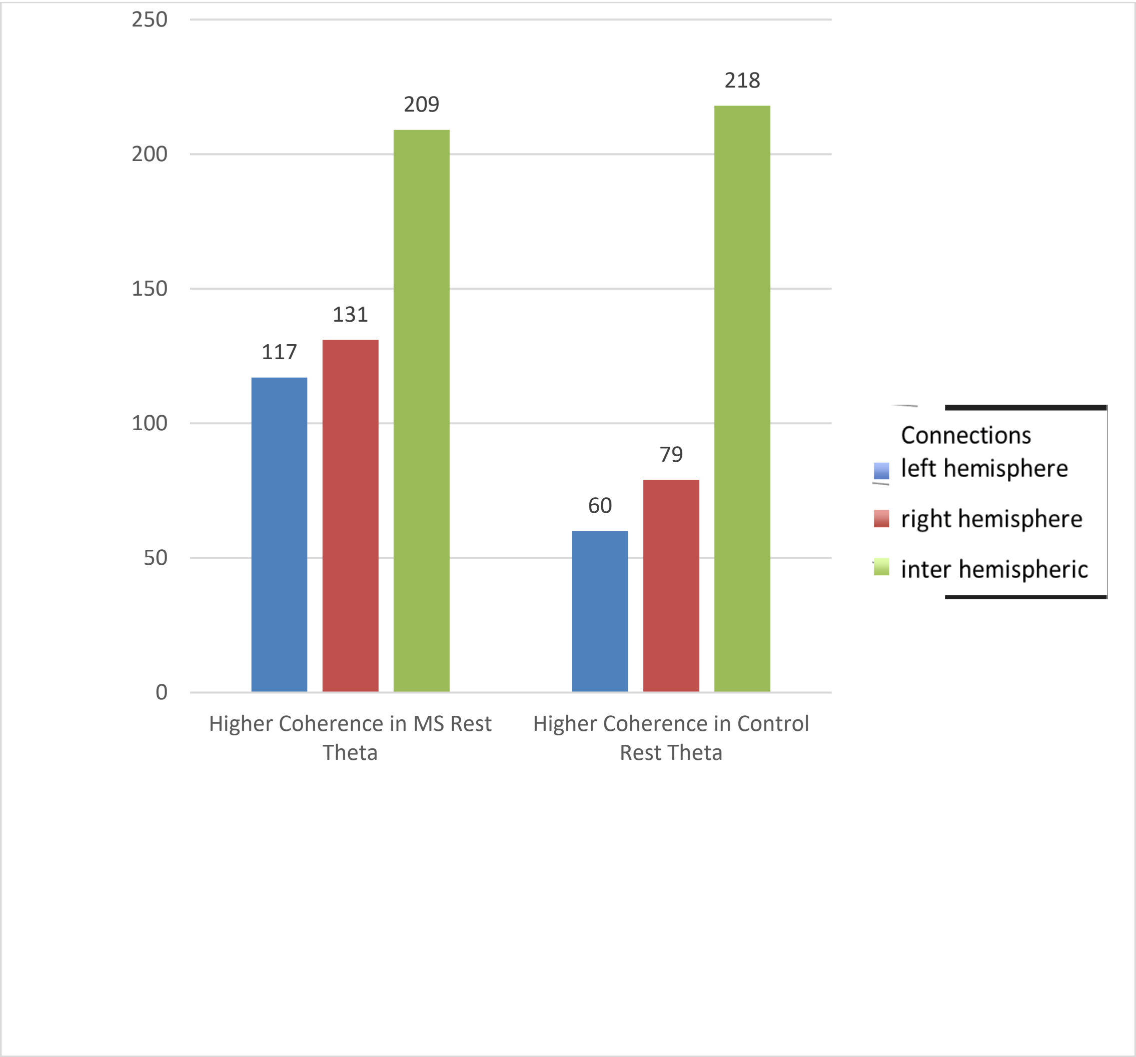

(B)

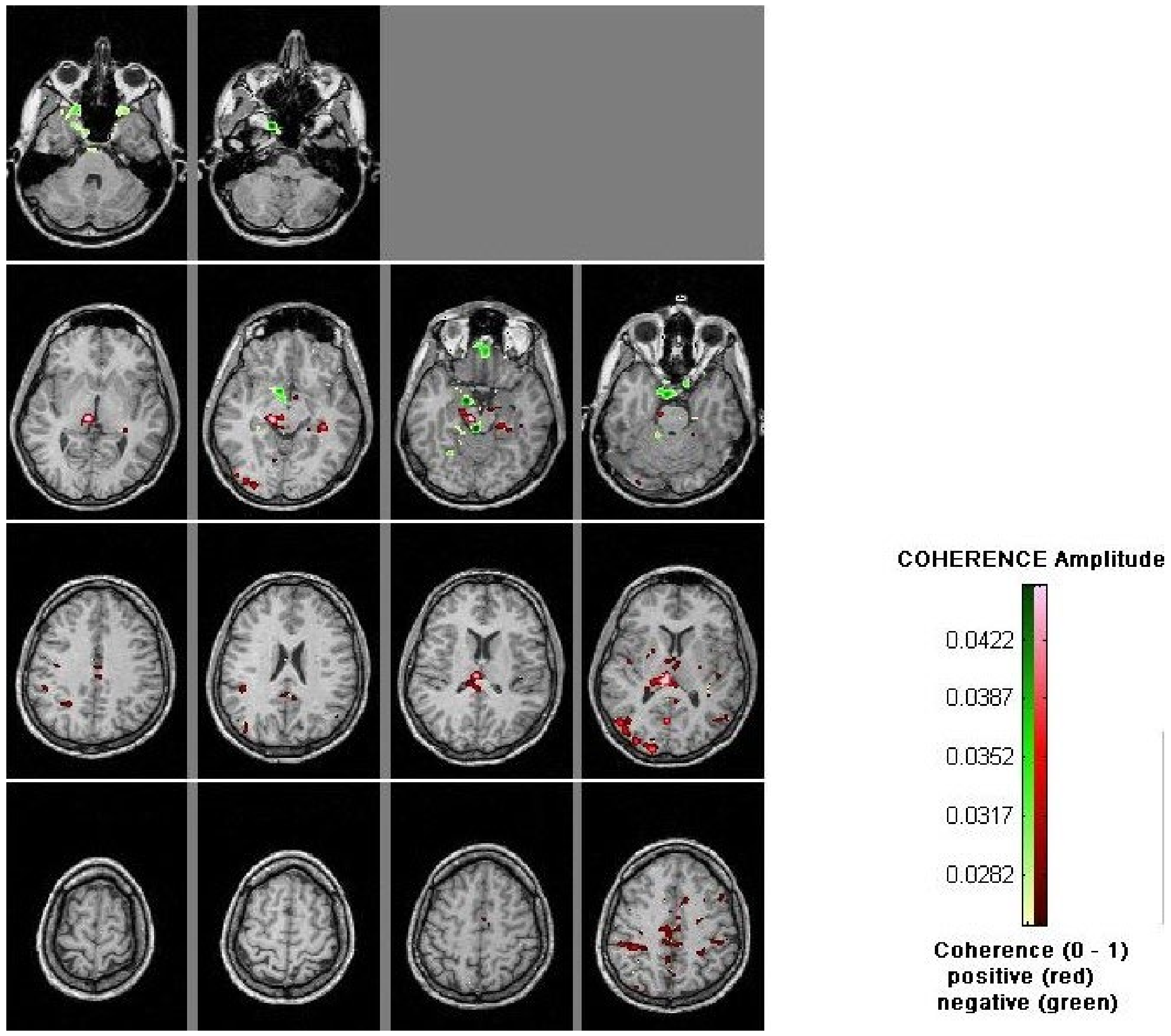

(C)

	Frontal	Temporal	Parietal	Cingulate	Occipital	Hippocampus	Fusiform	Insular	Striatum	Supramarginal
Frontal	12	16	66		32	2				
Temporal	16	3	52	5	24	1			7	
Parietal	66	52	38	19	65	9		10	26	
Cingulate		5	19	1	5		11	2	4	
Occipital	32	24	65	5	16	1		0	8	
Hippocampus	2	1	9		1	0		0	0	
Fusiform				11				3	16	
Insular			10	2	0	0	3		0	
Striatum		7	26	4	8	0	16	0		1
Supramarginal									1	

(D)

	Frontal	Temporal	Parietal	Cingulate	Occipital	Hippocampus	Fusiform	Insular	Striatum	Supramarginal
Frontal	46	75	18		32	13				
Temporal	75	28	4	2	25	15		11	16	
Parietal	18	4	0	0	0	4		1	1	
Cingulate		2	0	0	1		7	0	5	
Occipital	32	25	0	1	0	10		6	0	
Hippocampus	13	15	4		10	1		3	5	
Fusiform				7				10	17	
Insular		11	1	0	6	3	10		1	
Striatum		16	1	5	0	5	17	1		0
Supramarginal									0	

Supplementary Figure S1. Resting-state theta band connectivity differences between MS patients and HCs. (A) Spatial distribution of theta band connections on MRI; green indicates higher coherence in HC, red in MS; only differences between groups are shown. (B) Hemispheric connectivity counts comparison between groups. (C) Adjacency matrix of region-to-region connectivity in MS patients. (D) Adjacency matrix of region-to-region connectivity in healthy controls.
