# Supplementary material for: Functional Connectivity at Rest and During Cognitive Processing in Multiple Sclerosis: A Pilot Magnetoencephalography Study and Systematic Review
Source: Brain Sci. 2026 Aug 14;16(8):862. doi: 10.3390/brainsci16080862 (PMC13511113; doi:10.3390/brainsci16080862)
Supplement: Supplementary file 1 [file brainsci-16-00862-s001.zip › Supplementary figures/Supplementary Figure S2, Alpha band - resting state.pdf]

(A)

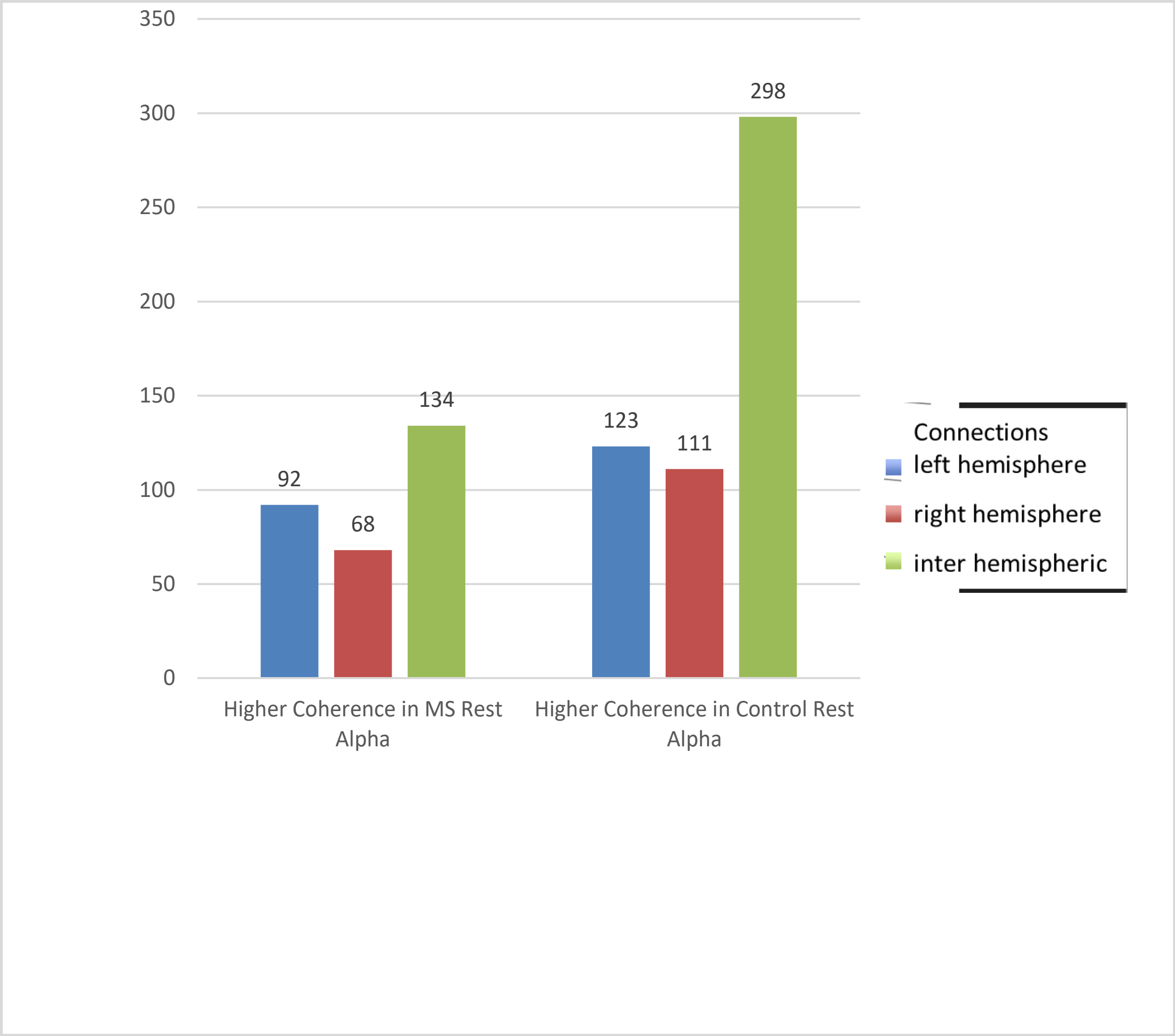

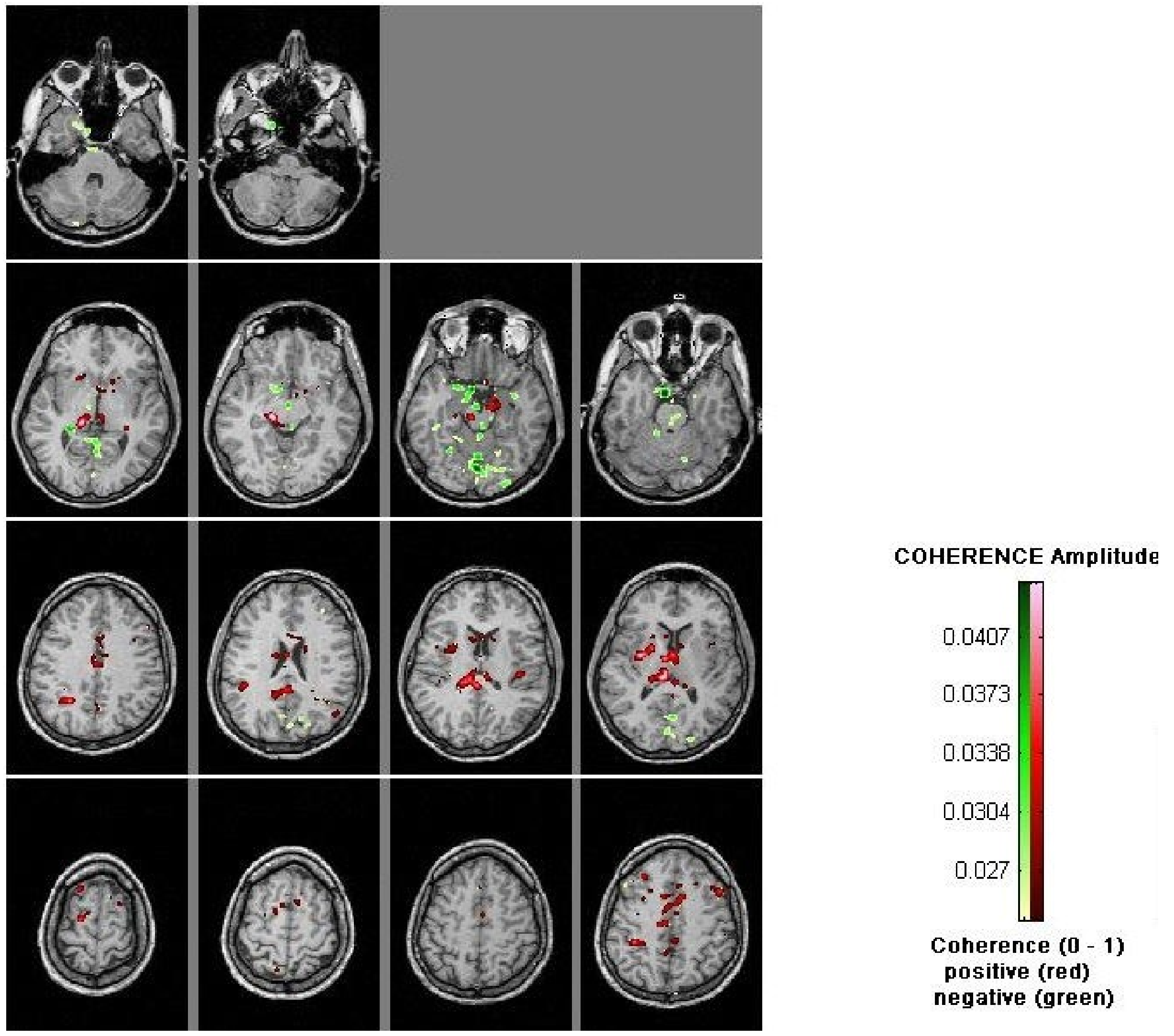

(B)

(C)

	Frontal	Temporal	Parietal	Cingulate	Occipital	Hippocampus	Fusiform	Insular	Striatum	Supramarginal
Frontal	34	19	50		15					
Temporal	19	0	11	9	0	0		0	14	
Parietal	50	11	11	11	7	0		5	26	
Cingulate		9	11	1	1		19	2	8	
Occipital	15	0	7	1	0	0		0	7	
Hippocampus		0	0		0	0		0	1	
Fusiform				19				6	32	
Insular		0	5	2	0	0	6		2	
Striatum		14	26	8	7	1	32	2		3
Supramarginal									3	

(D)

	Frontal	Temporal	Parietal	Cingulate	Occipital	Hippocampus	Fusiform	Insular	Striatum	Supramarginal
Frontal	8	49	25		58	11				
Temporal	49	41	37	2	88	17		13	2	
Parietal	25	37	7	0	53	12		3	1	
Cingulate		2	0	0	8		0	0	0	
Occipital	58	88	53	8	43	20		11	8	
Hippocampus	11	17	12		20	1		4	1	
Fusiform				0				8	1	
Insular		13	3	0	11	4	8			
Striatum		2	1	0	8	1	1			0
Supramarginal									0	

Supplementary Figure S2. Resting-state alpha band connectivity differences between MS patients and HC. (A) Spatial distribution of alpha band connections on MRI; green indicates higher coherence in HC, red in MS; only differences between groups are shown. (B) Hemispheric connectivity counts comparison between groups. (C) Adjacency matrix of region-to-region connectivity in MS patients. (D) Adjacency matrix of region-to-region connectivity in healthy controls.
