# Supplementary material for: Functional Connectivity at Rest and During Cognitive Processing in Multiple Sclerosis: A Pilot Magnetoencephalography Study and Systematic Review
Source: Brain Sci. 2026 Aug 14;16(8):862. doi: 10.3390/brainsci16080862 (PMC13511113; doi:10.3390/brainsci16080862)
Supplement: Supplementary file 1 [file brainsci-16-00862-s001.zip › Supplementary figures/Supplementary Figure S3, Beta band - resting state.pdf]

(A)

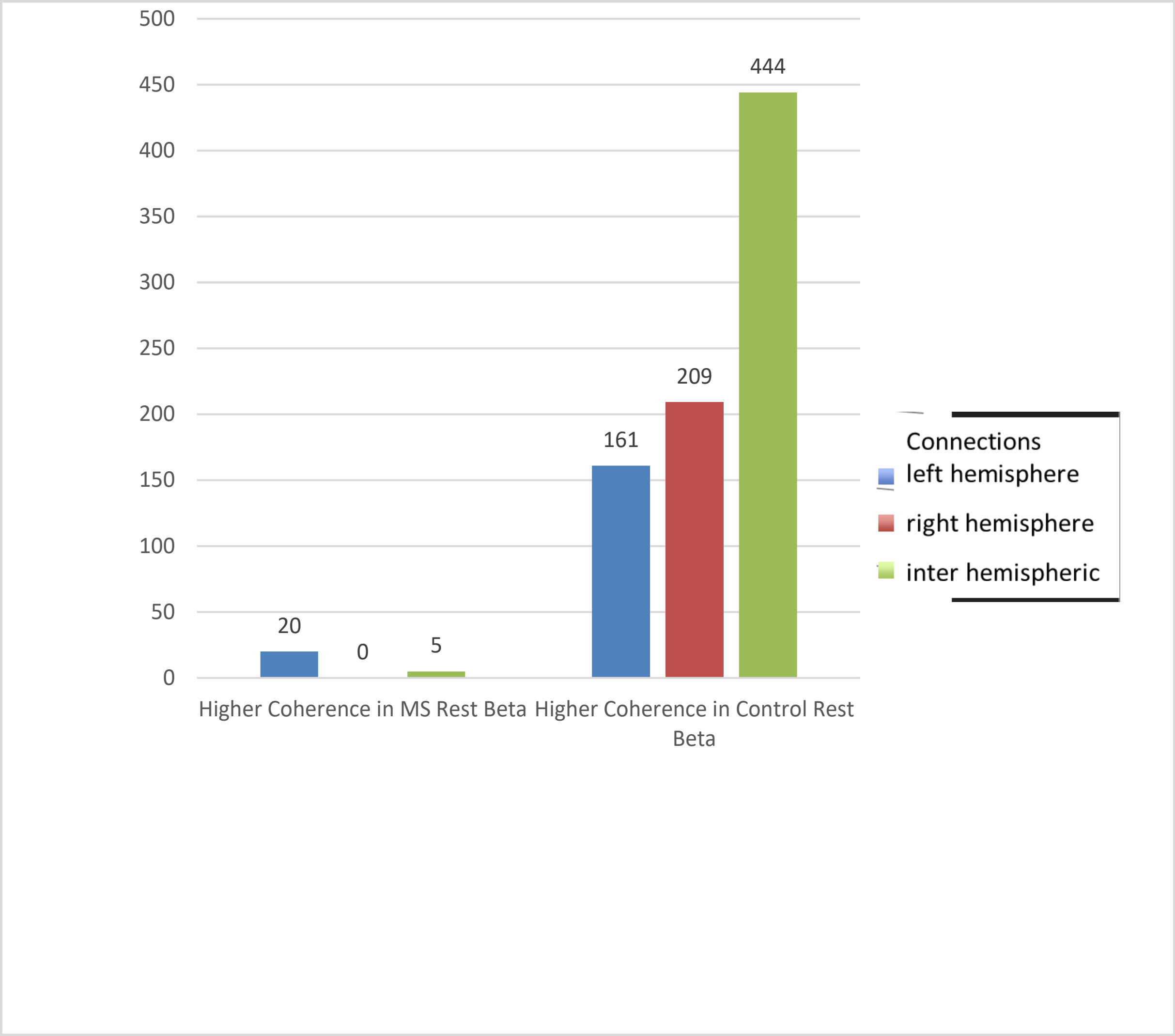

(B)

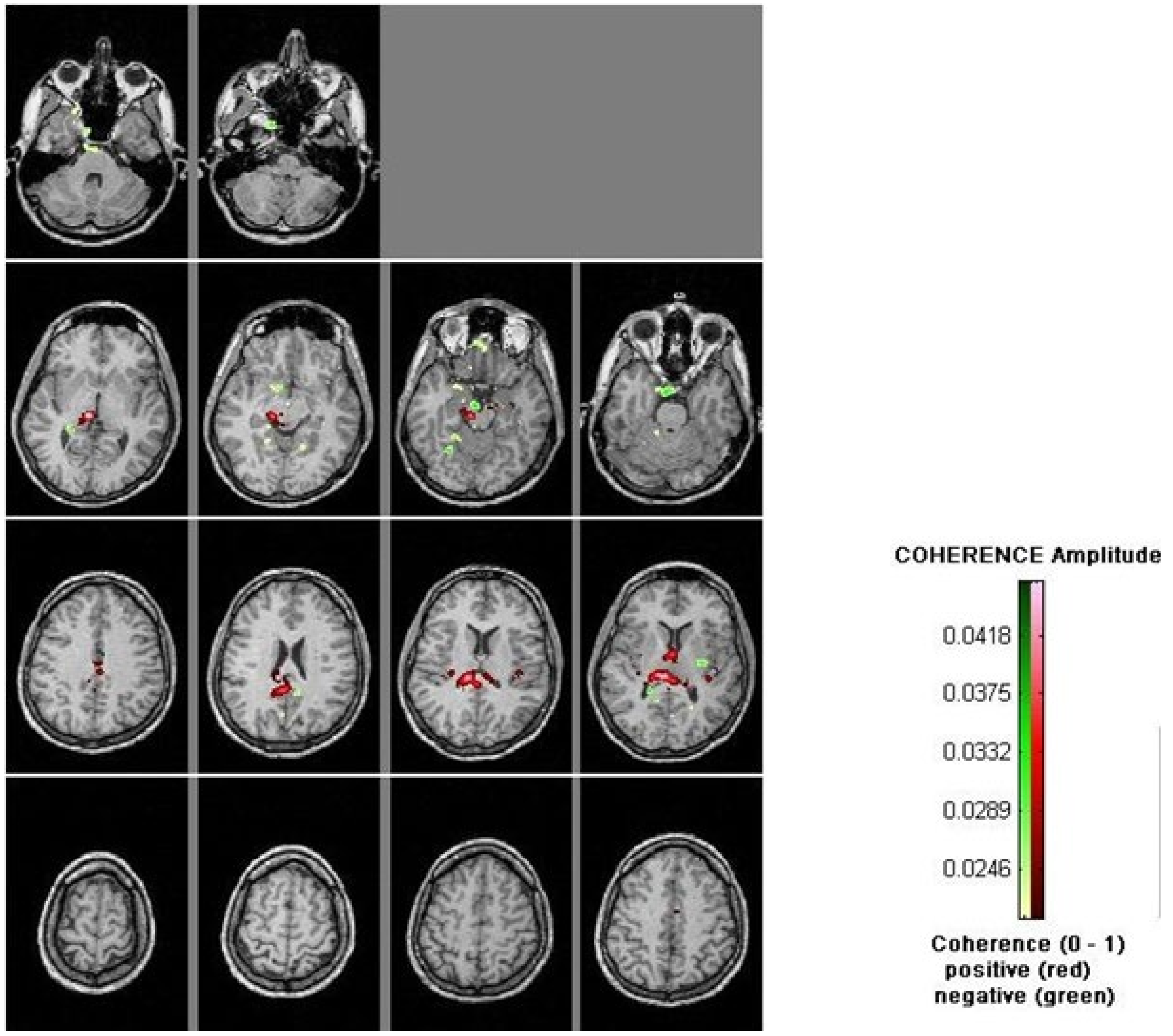

(C)

	Frontal	Temporal	Parietal	Cingulate	Occipital	Hippocampus	Fusiform	Insular	Striatum	Supramarginal
Frontal	0	1	2		1	0				
Temporal	1	0	0	1	0	0			1	
Parietal	2	0	0	1	0	0		0	3	
Cingulate		1	1	1	0	1	7	0	3	
Occipital	1	0	0	0	0	0		0	0	
Hippocampus	0	0	0	1	0	0	0	0	0	
Fusiform				7		0		0	3	
Insular			0	0	0	0	0	0	0	
Striatum		1	3	3	0	0	3	0		0
Supramarginal									0	

(D)

	Frontal	Temporal	Parietal	Cingulate	Occipital	Hippocampus	Fusiform	Insular	Striatum	Supramarginal
Frontal	57	113	81		76	18				
Temporal	113	42	69	4	71	17		16	25	
Parietal	81	69	14		53	12		10	10	
Cingulate		4			4		7			
Occipital	76	71	53	4	23	16		14	16	
Hippocampus	18	17	12		16	1		2	2	
Fusiform				7				18	19	
Insular		16	10		14	2	18	1	3	
Striatum		25	10		16	2	19	3		
Supramarginal										

Supplementary Figure S3. Resting-state beta band connectivity differences between MS patients and HC. (A) Spatial distribution of beta band connections on MRI; green indicates higher coherence in HC, red in MS; only differences between groups are shown. (B) Hemispheric connectivity counts comparison between groups. (C) Adjacency matrix of region-to-region connectivity in MS patients. (D) Adjacency matrix of region-to-region connectivity in healthy controls.
