# Supplementary material for: Functional Connectivity at Rest and During Cognitive Processing in Multiple Sclerosis: A Pilot Magnetoencephalography Study and Systematic Review
Source: Brain Sci. 2026 Aug 14;16(8):862. doi: 10.3390/brainsci16080862 (PMC13511113; doi:10.3390/brainsci16080862)
Supplement: Supplementary file 1 [file brainsci-16-00862-s001.zip › Supplementary figures/Supplementary Figure S4, gamma band - resting state.pdf]

(A)

(B)

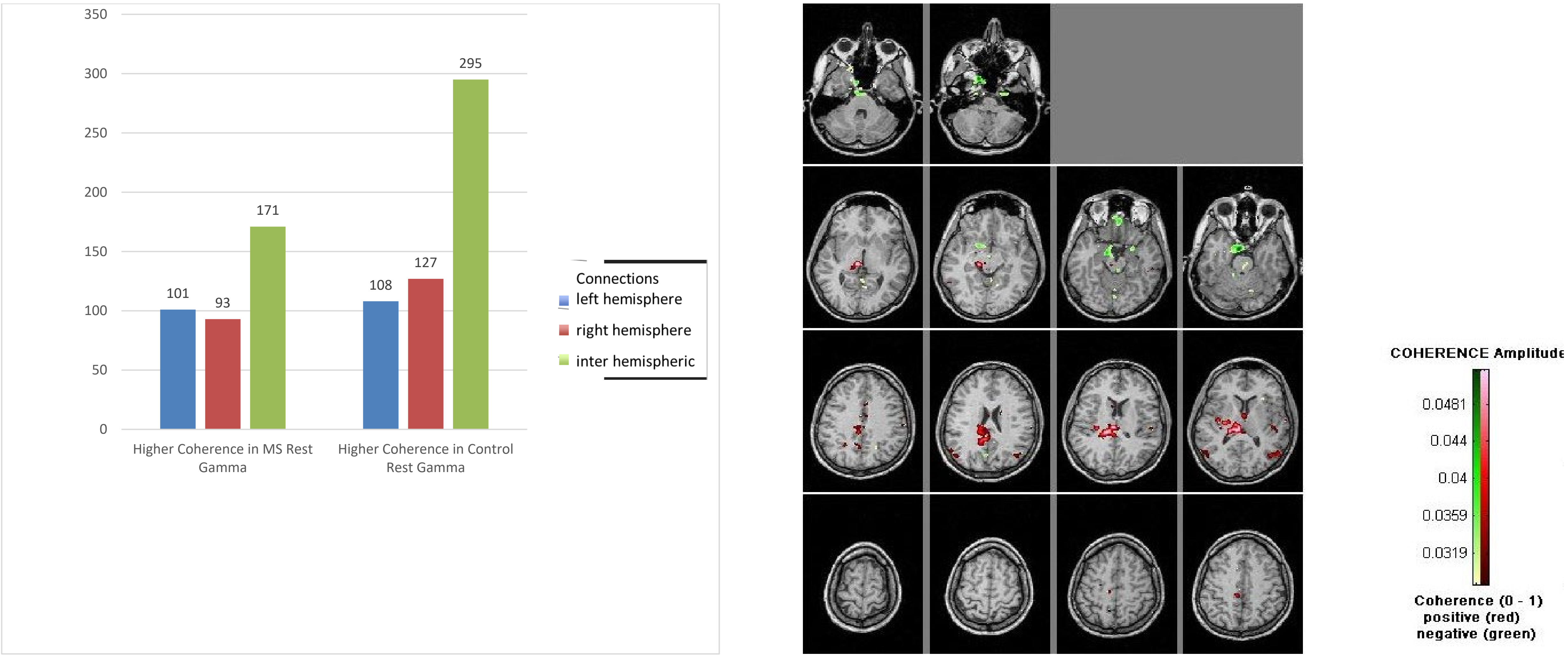

(C)

	Frontal	Temporal	Parietal	Cingulate	Occipital	Hippocampus	Fusiform	Insular	Striatum	Supramarginal
Frontal	28	28	66		11	1				
Temporal	28	3	45	5	12	0		1	2	
Parietal	66	45	31	13	41	2		5	20	
Cingulate		5	13	1	0	0	16	2	5	
Occipital	11	12	41	0	0	0		0	2	
Hippocampus	1	0	2	0	0	0	0	0	0	
Fusiform				16		0		3	21	
Insular		1	5	2	0	0	3	0	0	
Striatum		2	20	5	2	0	21	0		1
Supramarginal									1	

(D)

	Frontal	Temporal	Parietal	Cingulate	Occipital	Hippocampus	Fusiform	Insular	Striatum	Supramarginal
Frontal	39	73	30		57	20				
Temporal	73	31	24	3	58	18		15	18	
Parietal	30	24	1	0	14	8		3	3	
Cingulate		3	0	0	4	0	5	0	0	
Occipital	57	58	14	4	32	18		11	10	
Hippocampus	20	18	8	0	18	1	0	3	5	
Fusiform				5		0		7	12	
Insular		15	3	0	11	3	7	1	4	
Striatum		18	3	0	10	5	12	4		2
Supramarginal									2	

Supplementary Figure S4. Resting-state gamma band connectivity differences between MS patients and healthy controls. (A) Spatial distribution of gamma band connections on MRI; green indicates higher coherence in HC, red in MS; only differences between groups are shown. (B) Hemispheric connectivity counts comparison between groups. (C) Adjacency matrix of region-to-region connectivity in MS patients. (D) Adjacency matrix of region-to-region connectivity in healthy controls.
