# Supplementary material for: Functional Connectivity at Rest and During Cognitive Processing in Multiple Sclerosis: A Pilot Magnetoencephalography Study and Systematic Review
Source: Brain Sci. 2026 Aug 14;16(8):862. doi: 10.3390/brainsci16080862 (PMC13511113; doi:10.3390/brainsci16080862)
Supplement: Supplementary file 1 [file brainsci-16-00862-s001.zip › Supplementary figures/Supplementary Figure S5, Theta band - SDMT.pdf]

(A)

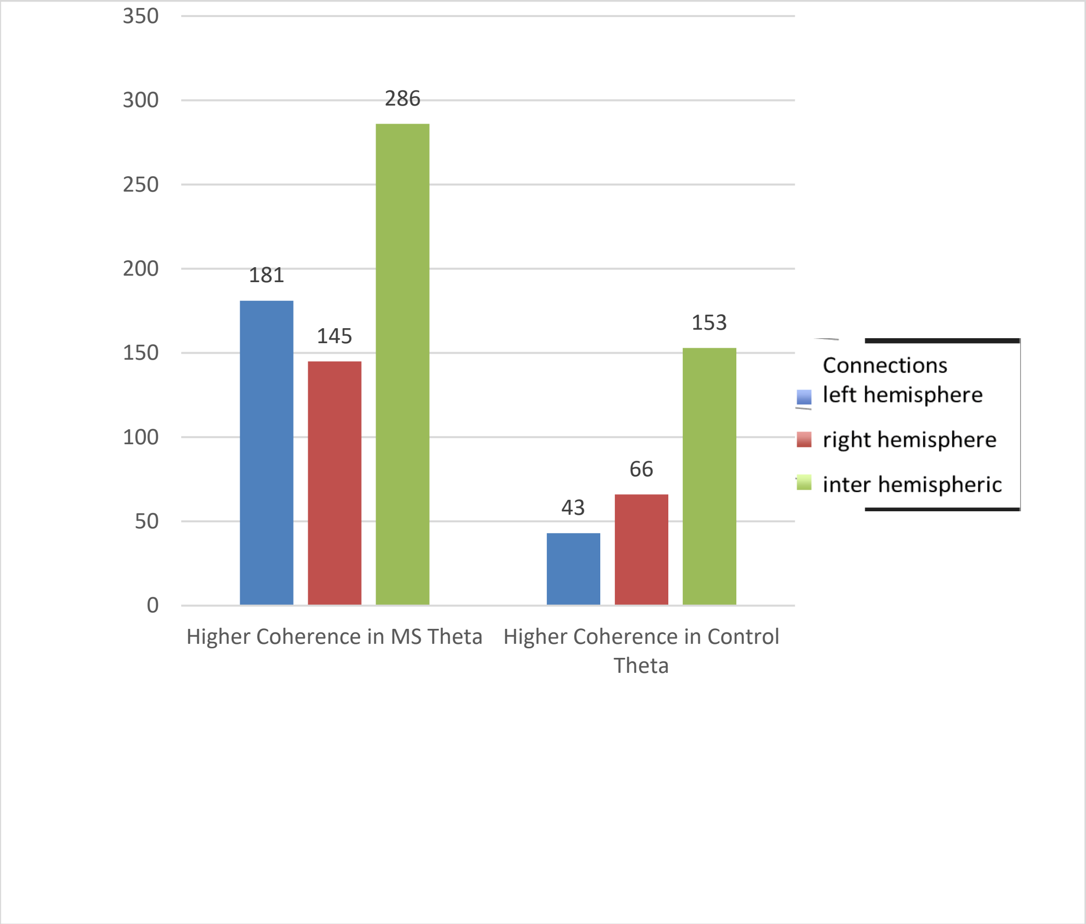

(B)

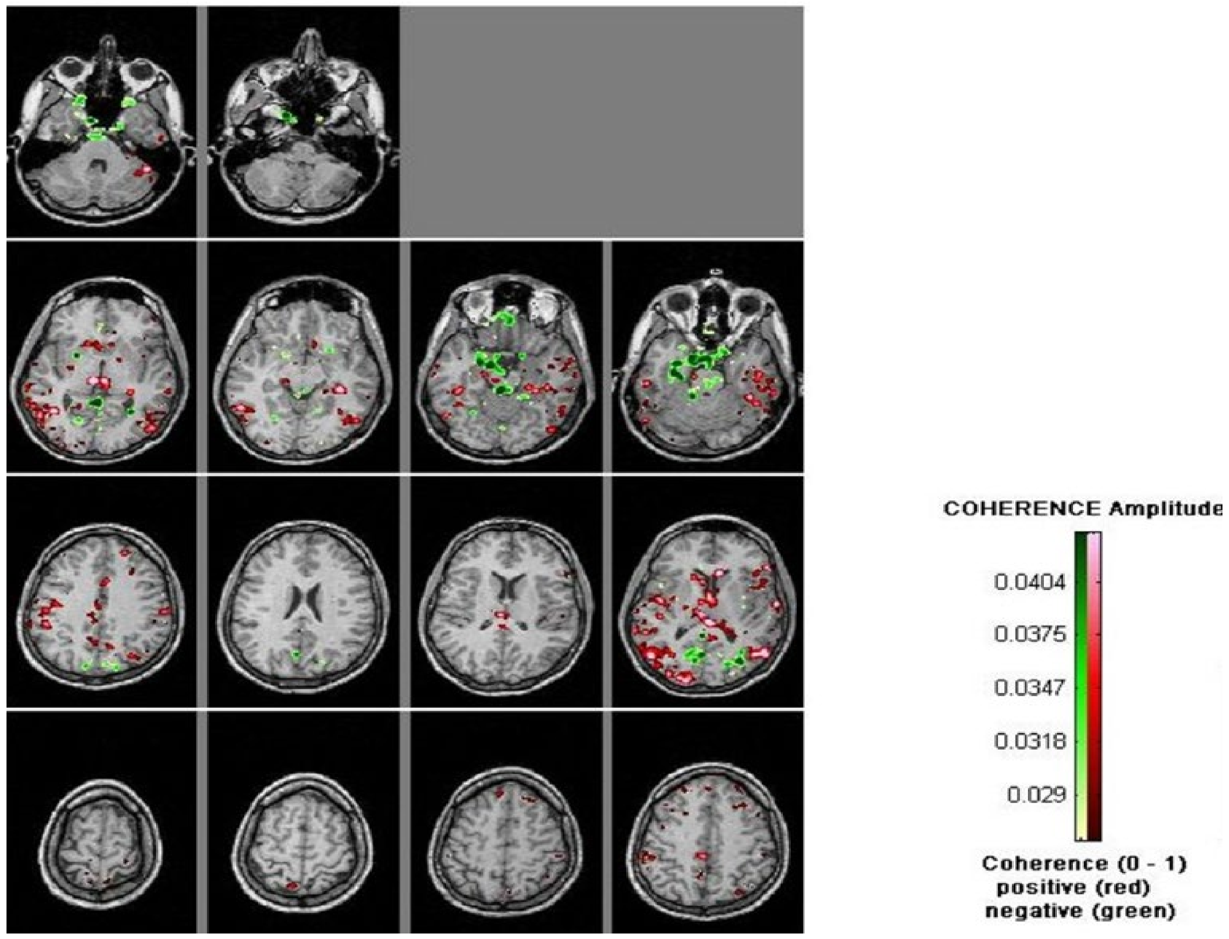

(C)

	Frontal	Temporal	Parietal	Cingulate	Occipital	Hippocampus	Fusiform	Insular	Striatum	Supramarginal
Frontal	21	39	92		82	2				
Temporal	39	14	65	1	51	1		4	9	
Parietal	92	65	38	8	70	11		9	23	
Cingulate		1	8	0	0	0	4	0	3	
Occipital	82	51	70	0	28	3		4	8	
Hippocampus	2	1	11	0	3	0	0	0	0	
Fusiform				4		0		3	18	
Insular		4	9	0	4	0	3	0	0	
Striatum		9	23	3	8	0	18	0		1
Supramarginal									1	

(D)

	Frontal	Temporal	Parietal	Cingulate	Occipital	Hippocampus	Fusiform	Insular	Striatum	Supramarginal
Frontal	33	58	2		9	12				
Temporal	58	25	3	3	15	13		12	9	
Parietal	2	3	0	1	1	4		3	2	
Cingulate		3	1	0	1	0	6	2	0	
Occipital	9	15	1	1	2	10		7	3	
Hippocampus	12	13	4	0	10	1	0	4	2	
Fusiform				6		0		11	5	
Insular		12	3	2	7	4	11	0	2	
Striatum		9	2	0	3	2	5	2		1
Supramarginal									1	

Supplementary Figure S5. Theta band connectivity differences during SDMT performance between MS patients and healthy controls. (A) Task-related theta band connectivity displayed on MRI; green indicates higher coherence in HC, red in MS; only differences between groups are shown. (B) Hemispheric connectivity counts during SDMT. (C) Adjacency matrix of region-to-region connectivity in MS patients during task performance. (D) Adjacency matrix of region-to-region connectivity in healthy controls during task performance.
