# Supplementary material for: Functional Connectivity at Rest and During Cognitive Processing in Multiple Sclerosis: A Pilot Magnetoencephalography Study and Systematic Review
Source: Brain Sci. 2026 Aug 14;16(8):862. doi: 10.3390/brainsci16080862 (PMC13511113; doi:10.3390/brainsci16080862)
Supplement: Supplementary file 1 [file brainsci-16-00862-s001.zip › Supplementary figures/Supplementary Figure S6, Alpha band - SDMT.pdf]

(A)

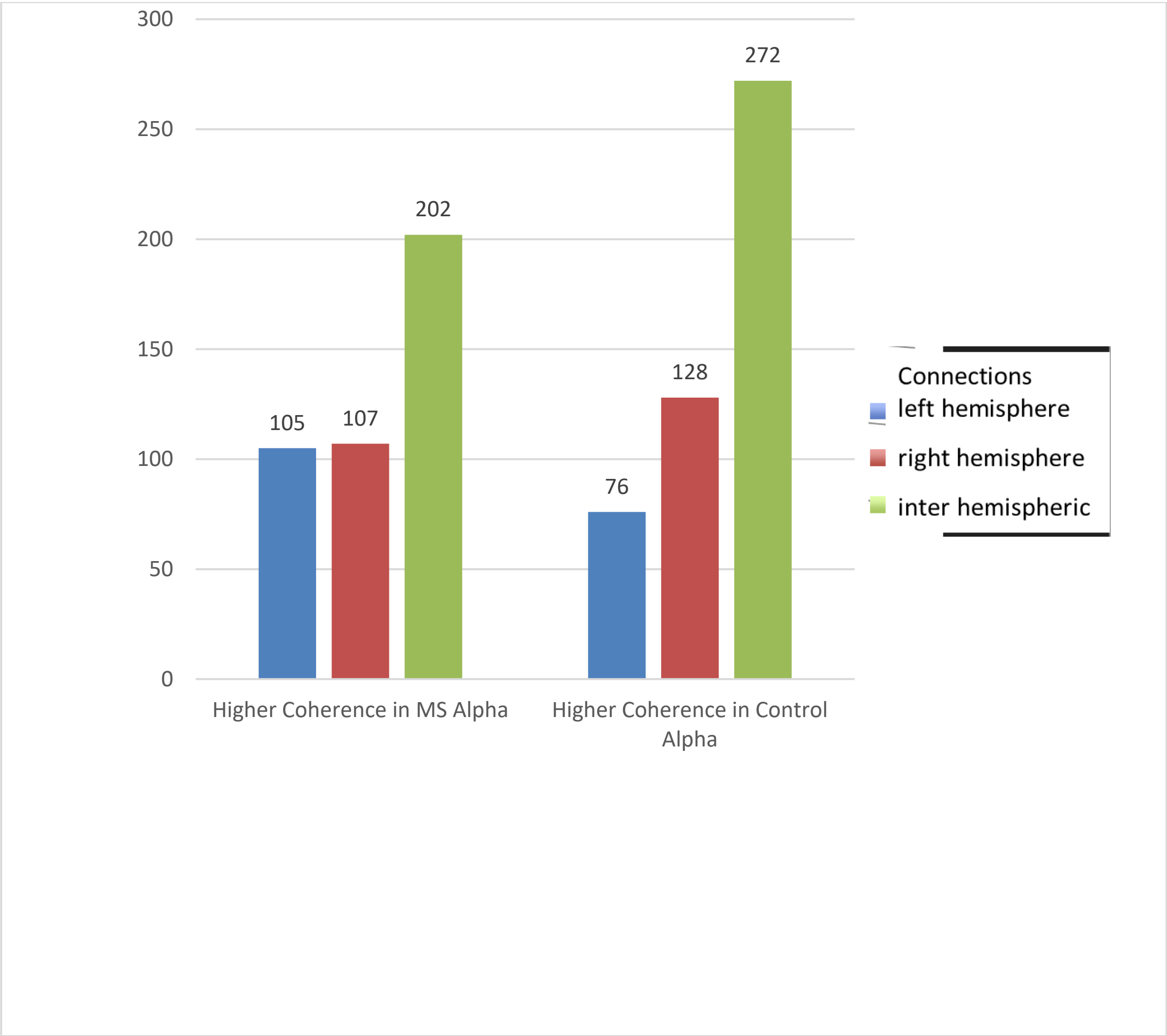

(B)

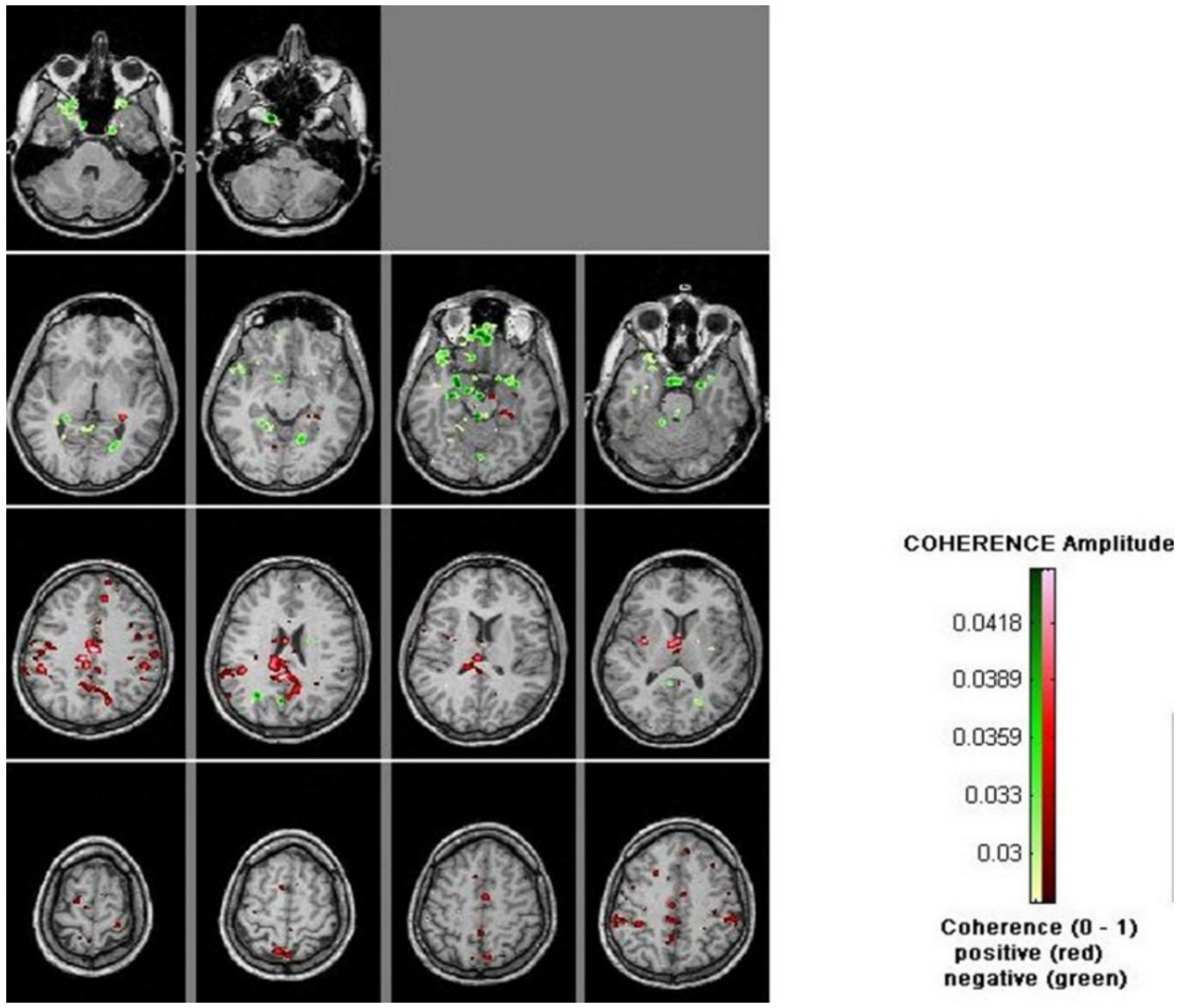

(C)

	Frontal	Temporal	Parietal	Cingulate	Occipital	Hippocampus	Fusiform	Insular	Striatum	Supramarginal
Frontal	15	14	78		38	0				
Temporal	14	0	52	6	15	0		0	5	
Parietal	78	52	34	11	59	2		6	18	
Cingulate		6	11	1	5	0	13	0	5	
Occipital	38	15	59	5	15	0		0	7	
Hippocampus	0	0	2	0	0	0	0	0	0	
Fusiform				13		0		1	13	
Insular		0	6	0	0	0	1		0	
Striatum		5	18	5	7	0	13	0		1
Supramarginal									1	

(D)

	Frontal	Temporal	Parietal	Cingulate	Occipital	Hippocampus	Fusiform	Insular	Striatum	Supramarginal
Frontal	51	85	17		50	18				
Temporal	85	35	4	3	42	19		16	17	
Parietal	17	4	0	1	3	9		2	8	
Cingulate		3	1	0	3	0	5	0	0	
Occipital	50	42	3	3	5	17		10	11	
Hippocampus	18	19	9	0	17	1	0	4	3	
Fusiform				5		0		15	17	
Insular		16	2	0	10	4	15	1	3	
Striatum		17	8	0	11	3	17	3		1
Supramarginal									1	

Supplementary Figure S6. Alpha band connectivity differences during SDMT performance between MS patients and healthy controls. (A) Task-related alpha band connectivity displayed on MRI; green indicates higher coherence in HC, red in MS; only differences between groups are shown. (B) Hemispheric connectivity counts during SDMT. (C) Adjacency matrix of region-to-region connectivity in MS patients during task performance. (D) Adjacency matrix of region-to-region connectivity in healthy controls during task performance.
