# Supplementary material for: Functional Connectivity at Rest and During Cognitive Processing in Multiple Sclerosis: A Pilot Magnetoencephalography Study and Systematic Review
Source: Brain Sci. 2026 Aug 14;16(8):862. doi: 10.3390/brainsci16080862 (PMC13511113; doi:10.3390/brainsci16080862)
Supplement: Supplementary file 1 [file brainsci-16-00862-s001.zip › Supplementary figures/Supplementary Figure S7, Beta band - SDMT.pdf]

(A)

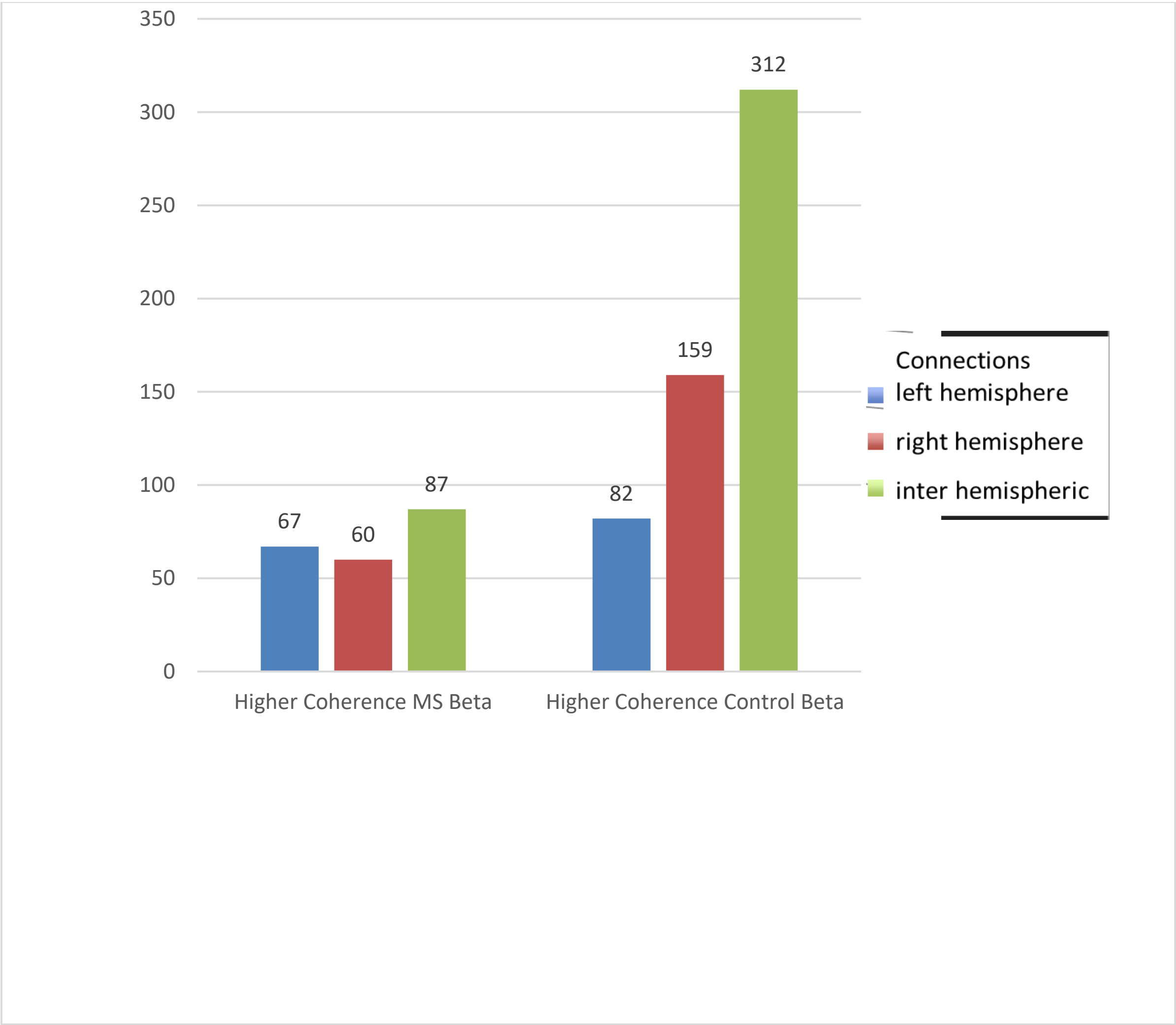

(B)

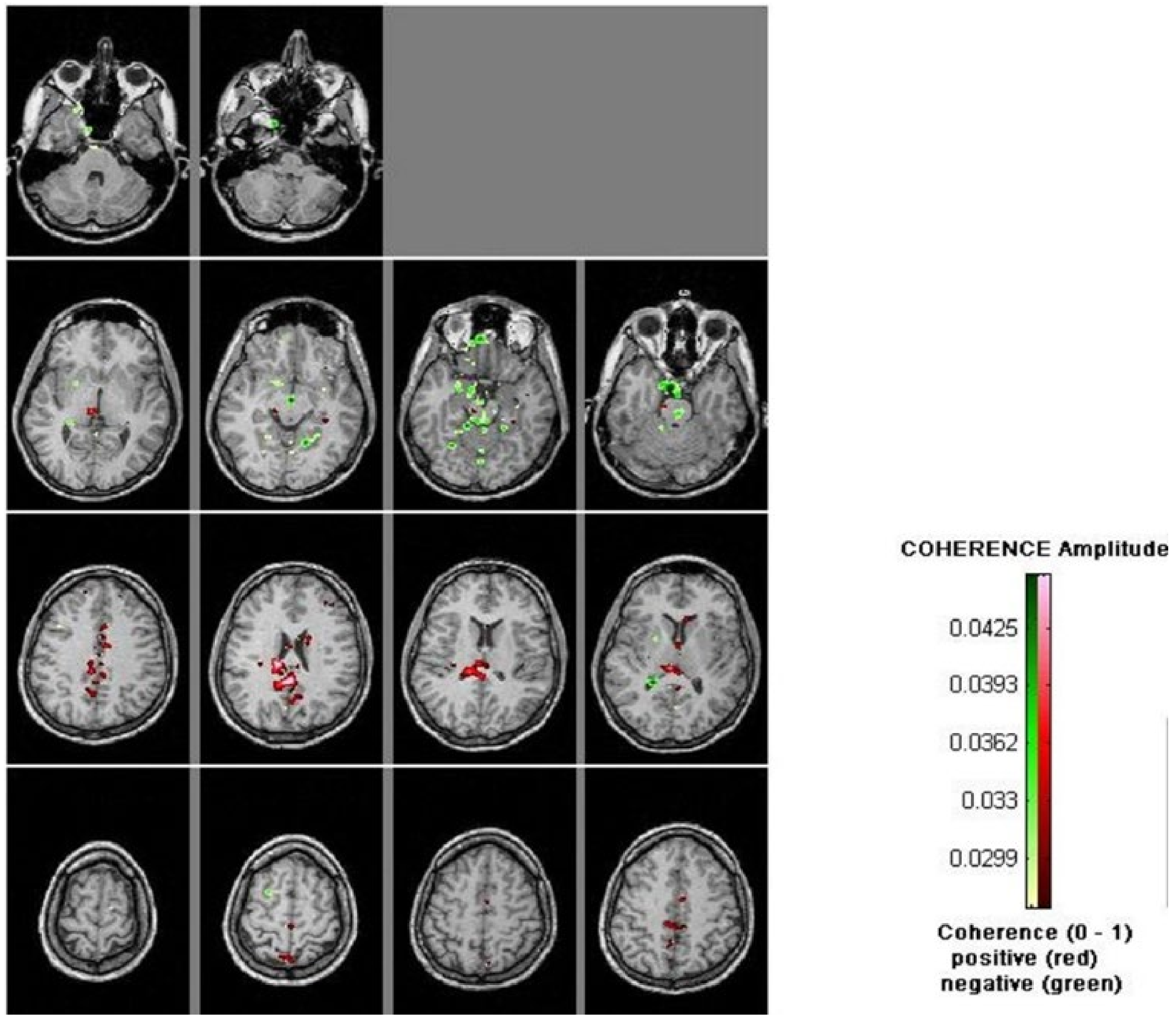

(C)

	Frontal	Temporal	Parietal	Cingulate	Occipital	Hippocampus	Fusiform	Insular	Striatum	Supramarginal
Frontal	3	4	6		28	0				
Temporal	4	1	2	4	18	0		0	6	
Parietal	6	2	2	5	35	0		0	16	
Cingulate		4	5	0	5	0	11	1	6	
Occipital	28	18	35	5	18	1		3	16	
Hippocampus	0	0	0	0	1	0	0	0	0	
Fusiform				11		0		0	19	
Insular		0	0	1	3	0	0	0	1	
Striatum		6	16	6	16	0	19	1		3
Supramarginal									3	

(D)

	Frontal	Temporal	Parietal	Cingulate	Occipital	Hippocampus	Fusiform	Insular	Striatum	Supramarginal
Frontal	64	105	46		39	0				
Temporal	105	39	42	8	35	16		13	13	
Parietal	46	42	0	0	7	10		6	8	
Cingulate		8	0	0	4	1	4	0	0	
Occipital	39	35	7	4	0	13		7	8	
Hippocampus	0	16	10	1	13	1	20	2	4	
Fusiform				4		20		19	11	
Insular		13	6	0	7	2	19	1	2	
Striatum		13	8	0	8	4	11	2		1
Supramarginal									1	

Supplementary Figure S7. Beta band connectivity differences during SDMT performance between MS patients and healthy controls. (A) Task-related beta band connectivity displayed on MRI; green indicates higher coherence in HC, red in MS; only differences between groups are shown. (B) Hemispheric connectivity counts during SDMT. (C) Adjacency matrix of region-to-region connectivity in MS patients during task performance. (D) Adjacency matrix of region-to-region connectivity in healthy controls during task performance.
