# Supplementary material for: Functional Connectivity at Rest and During Cognitive Processing in Multiple Sclerosis: A Pilot Magnetoencephalography Study and Systematic Review
Source: Brain Sci. 2026 Aug 14;16(8):862. doi: 10.3390/brainsci16080862 (PMC13511113; doi:10.3390/brainsci16080862)
Supplement: Supplementary file 1 [file brainsci-16-00862-s001.zip › Supplementary figures/Supplementary Figure S8, gamma band - SDMT.pdf]

(A)

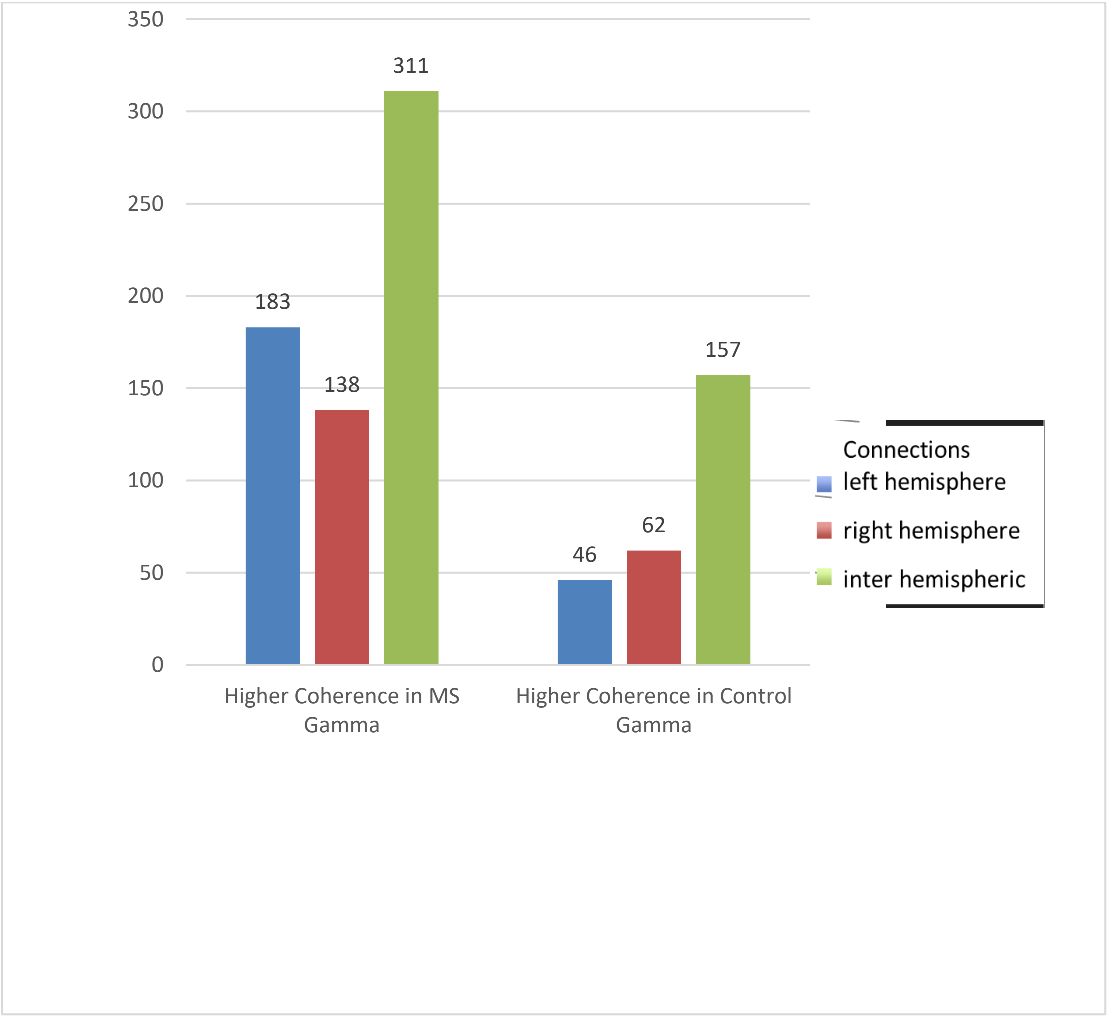

(B)

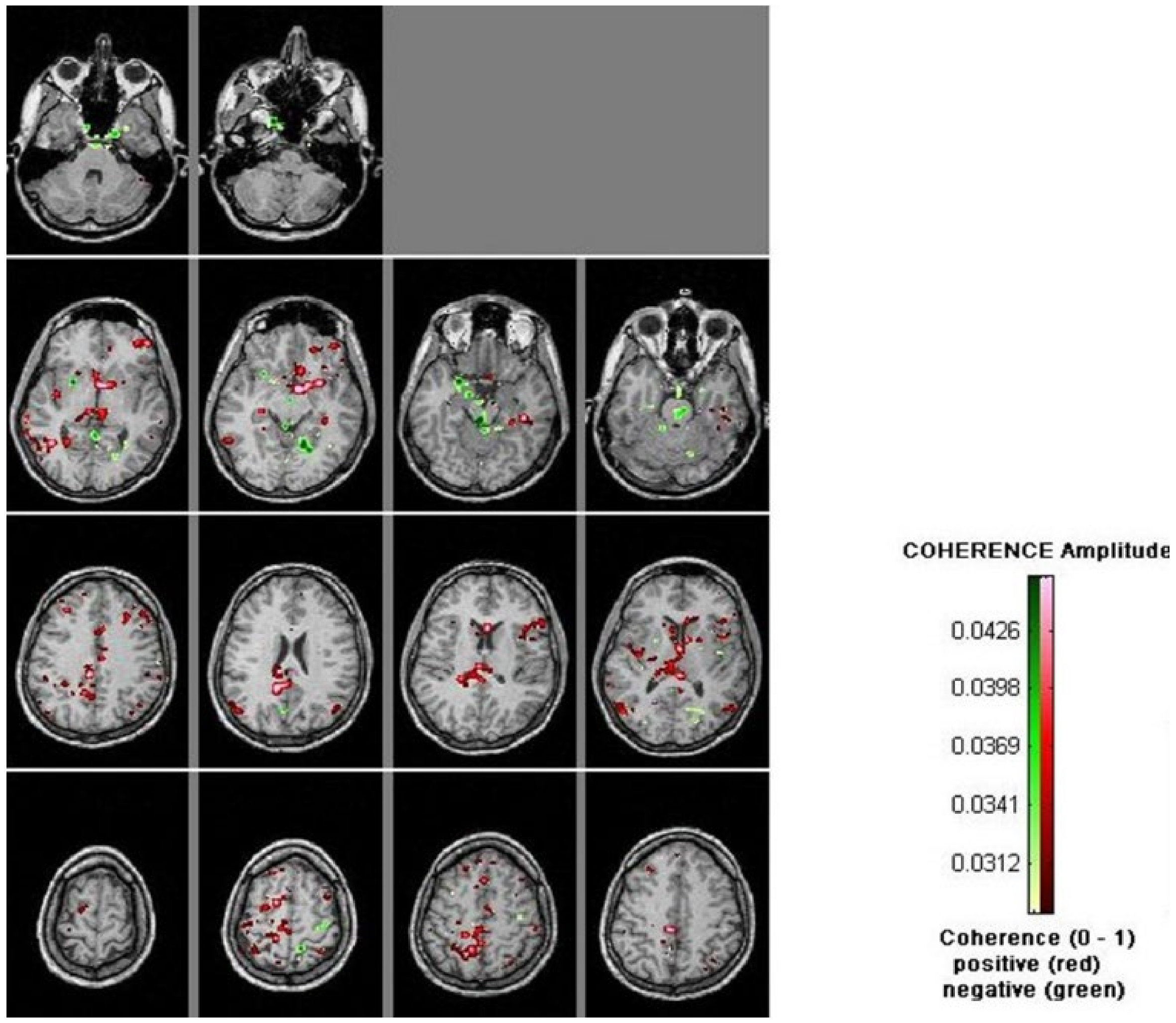

(C)

	Frontal	Temporal	Parietal	Cingulate	Occipital	Hippocampus	Fusiform	Insular	Striatum	Supramarginal
Frontal	42	48	85		55	6				
Temporal	48	3	61	8	19	0		1	11	
Parietal	85	61	37	8	59	12		14	31	
Cingulate		8	8	1	5	3	17	4	8	
Occipital	55	19	59	5	14	1		4	12	
Hippocampus	6	0	12	3	1	0	0	0	2	
Fusiform				17		0		16	38	
Insular		1	14	4	4	0	16	0	3	
Striatum		11	31	8	12	2	38	3		4
Supramarginal									4	

(D)

	Frontal	Temporal	Parietal	Cingulate	Occipital	Hippocampus	Fusiform	Insular	Striatum	Supramarginal
Frontal	8	48	8		26	12				
Temporal	48	32	8	0	35	19		9	5	
Parietal	8	8	0	0	6	4		2	2	
Cingulate		0	0	0	2		0	0	0	
Occipital	26	35	6	2	7	10		6	7	
Hippocampus	12	19	4		10	1		3	1	
Fusiform				0				3	0	
Insular		9	2	0	6	3	3		1	
Striatum		5	2	0	7	1	0	1		0
Supramarginal									0	

Supplementary Figure S8. Gamma band connectivity differences during SDMT performance between MS patients and healthy controls. (A) Task-related gamma band connectivity displayed on MRI; green indicates higher coherence in HC, red in MS; only differences between groups are shown. (B) Hemispheric connectivity counts during SDMT. (C) Adjacency matrix of region-to-region connectivity in MS patients during task performance. (D) Adjacency matrix of region-to-region connectivity in healthy controls during task performance.
