# Supplementary material for: Functional Connectivity at Rest and During Cognitive Processing in Multiple Sclerosis: A Pilot Magnetoencephalography Study and Systematic Review
Source: Brain Sci. 2026 Aug 14;16(8):862. doi: 10.3390/brainsci16080862 (PMC13511113; doi:10.3390/brainsci16080862)
Supplement: Supplementary file 1 [file brainsci-16-00862-s001.zip › Supplementary tables/Supplementary Table S1, search strategy.pdf]

Supplementary Table S1: Full search strategies for each database, including all controlled vocabulary and free-text terms.

**Appendix A**: Databases were used to search for articles related to the following keywords:		
**Databases**	**Search Strategy**	**Results**
**Pubmed**	("Multiple Sclerosis"[MeSH Terms] OR "Multiple Sclerosis"[Title/Abstract]) AND ("Magnetoencephalography"[MeSH Terms] OR "Magnetoencephalography"[Title/Abstract] OR "MEG"[Title/Abstract] OR "magneto-encephalography"[Title/Abstract] OR "oscillation*"[Title/Abstract])	210
**Scopus**	TITLE-ABS-KEY ("Multiple Sclerosis") AND TITLE-ABS-KEY ("Magnetoencephalography" OR "MEG" OR "magneto-encephalography" OR "oscillation*")	378
**Web of Science**	TS=("Multiple Sclerosis") AND TS=("Magnetoencephalography" OR "MEG" OR "magneto-encephalography" OR "oscillation*")	338
**Cochrane library**	([mh "Multiple Sclerosis"] OR "Multiple Sclerosis":ti,ab,kw) AND ([mh "Magnetoencephalography"] OR "Magnetoencephalography":ti,ab,kw OR "MEG":ti,ab,kw OR "magneto-encephalography":ti,ab,kw OR "oscillation*":ti,ab,kw)	28
