# Supplementary material for: Functional Connectivity at Rest and During Cognitive Processing in Multiple Sclerosis: A Pilot Magnetoencephalography Study and Systematic Review
Source: Brain Sci. 2026 Aug 14;16(8):862. doi: 10.3390/brainsci16080862 (PMC13511113; doi:10.3390/brainsci16080862)
Supplement: Supplementary file 1 [file brainsci-16-00862-s001.zip › Supplementary tables/Supplementary Table S3, Reasons for exclusion.pdf]

**Supplementary Table S3**: Full-text reports excluded from the systematic review (n = 11), with the specific reason for exclu-sion for each study

| Excluded Article | PICO Evaluation | Reason for Exclusion |
|---|---|---|
| Gu (2021) [1] | The population includes relapsing-remitting multiple sclerosis (RRMS) patients and healthy controls, satisfying the population and comparison parameters, but utilizes structural MRI and generalized cognitive scales (MoCA) instead of MEG-derived parameters. | Excluded due to incorrect outcomes, as the study does not assess cognitive tasks using MEG-derived parameters. |
| Hsu et al. (2023) [2] | The trial evaluates MS patients undergoing transcranial alternating current stimulation, but the primary outcome measures information processing speed via the Symbol Digit Modalities Test. | Excluded for lacking relevant MEG-derived parameters, focusing instead on processing speed and intervention tolerability. |
| Liang et al. (2023) [3] | The study analyzes acute and remitting MS patient data against healthy controls using a support vector machine model to evaluate resting-state fMRI amplitude fluctuations. | Excluded because the outcomes are strictly based on neuroimaging classification model performance and fail to evaluate MEG-derived parameters (Liang et al., 2023). |
| Linnhoff et al. (2023) [4] | The research includes MS population and healthy controls, matching P and C criteria, but evaluates fatigability-related EEG oscillatory brain activity during sustained attention tasks. | Excluded because the intervention and outcomes assess physical/mental exhaustion and reaction time variability rather than MEG-derived parameters. |
| Martí-Juan et al. (2022) [5] | The study analyzes structural and functional connectivity in MS patients via whole-brain models compared with SDMT and EDSS scores (Martí-Juan et al., 2022). | Excluded because the outcome metrics map generalized cognitive impairment and disability without targeting cognitive tasks using MEG-derived parameters. |
| Motolese et al. (2023) [6] | The trial involves MS patients and assesses psychological statuses such as perceived stress, rumination, depression, and fatigue using clinical questionnaires and EEG. | Excluded because the study evaluates broad psychological functioning and stress responses rather than specific cognitive functions using MEG-derived parameters. |

| | | |
|---|---|---|
| Riem et al. (2022) [7] | The study assesses MS patients and controls walking on a treadmill, focusing on step width, center of mass, and fall risks. | Excluded due to entirely biomechanical and motor function outcomes, containing no MEG or neuropsychological evaluations (Riem et al., 2022). |
| Aine (2014) [8] | The text discusses generalized magnetoencephalography (MEG) stimulus parameters and data analysis methodologies. | Excluded because it lacks an MS patient population, clinical intervention, and cognitive outcomes. |
| Anninos (2016) [9] | The document contains only raw numerical data arrays for MEG/EEG baseline runs without clinical context. | Excluded for lacking an identified MS population, comparative analysis, and specific cognitive outcome measures. |
| Capone et al. (2020) [10] | The study compares MS patients to healthy controls during fatiguing motor tasks by evaluating high-frequency oscillations and somatosensory evoked potentials. | Excluded because the research endpoints are purely neurophysiological and strength-based, remaining unrelated to cognition. |
| Figueroa-Vargas et al. (2020) [11] | The research evaluates early-stage RRMS patients and healthy controls during working memory encoding and maintenance tasks using EEG. | Excluded because it specifically tests visuospatial and verbal working memory rather than cognitive MEG-derived parameters. |

MS: Multiple Sclerosis; MoCA: Montreal Cognitive Assessment; SDMT: Symbol Digit Modalities Test; fMRI: Functional Magnetic Resonance Imaging; EEG: Electroencephalography; MEG: Magnetoencephalography; EDSS: Expanded Disability Status Scale; RRMS: Relapsing-Remitting Multiple Sclerosis; MRI: Magnetic Resonance Imaging; PICO: Population, Intervention, Comparison, Outcome

## REFERENCES


1. Gu X-Q, Liu Y, Gu J-B, Li L-F, Fu L-L, Han X-M: **Correlations between hippocampal functional connectivity, structural changes, and clinical data in patients with relapsing-remitting multiple sclerosis: a case-control study using multimodal magnetic resonance imaging**. *Neural Regeneration Research* 2022, **17**(5):1115-1124. **doi**: 10.4103/1673-5374.324855
2. Hsu W-Y, Zanto T, Park JE, Gazzaley A, Bove RM: **Effects of transcranial alternating current stimulation on cognitive function in people with multiple sclerosis: A randomized controlled trial**. *Multiple Sclerosis and Related Disorders* 2023, **80**:105090. **doi**: 10.1016/j.msard.2023.105090
3. Liang X, Wang L, Zhu Y, Wang Y, He T, Wu L, Huang M, Zhou F: **Altered neural intrinsic oscillations in patients with multiple sclerosis: effects of cortical thickness**. *Front Neurol* 2023, **14**:1143646. **doi**: 10.3389/fneur.2023.1143646
4. Linnhoff S, Haghikia A, Zaehle T: **Fatigability-related oscillatory brain activity changes in people with MS**. *Multiple Sclerosis and Related Disorders* 2023, **69**:104457. **doi**: 10.1016/j.msard.2022.104457
5. Martí-Juan G, Sastre-Garriga J, Martinez-Heras E, Vidal-Jordana A, Llufriu S, Groppa S, Gonzalez-Escamilla G, Rocca MA, Filippi M, Høgestøl EA *et al*: **Using The Virtual Brain to study the relationship between structural and functional connectivity in patients with multiple sclerosis: a multicentre study**. **doi**:
6. Motolese F, Stelitano D, Lanzone J, Albergo G, Cruciani A, Masciulli C, Musumeci G, Pilato F, Rossi M, Ribolsi M *et al*: **Feasibility and efficacy of an at-home, smart-device aided mindfulness program in people with Multiple Sclerosis**. *Multiple Sclerosis and Related Disorders* 2023, **78**:104931. **doi**: 10.1016/j.msard.2023.104931
7. Riem L, Beardsley SA, Obeidat AZ, Schmit BD: **Visual oscillation effects on dynamic balance control in people with multiple sclerosis**. *J NeuroEngineering Rehabil* 2022, **19**(1):90. **doi**: 10.1186/s12984-022-01060-0
8. Aine CJ, Supek S, Sanfratello L, Stephen JM: **Selection of Stimulus Parameters for Visual MEG Studies of Sensation and Cognition**. In: *Magnetoencephalography.* edn. Edited by Supek S, Aine CJ. Berlin, Heidelberg: Springer Berlin Heidelberg; 2014: 767-799.
9. Anninos P, Adamopoulos A, Kotini A, Tsagas N: **MEG evaluation of pico-Tesla external TMS on multiple sclerosis patients**. *Multiple Sclerosis and Related Disorders* 2016, **8**:45-53. **doi**: 10.1016/j.msard.2016.04.008
10. Capone F, Motolese F, Rossi M, Musumeci G, Insola A, Di Lazzaro V: **Thalamo-cortical dysfunction contributes to fatigability in multiple sclerosis patients: A neurophysiological study**. *Multiple Sclerosis and Related Disorders* 2020, **39**:101897. **doi**: 10.1016/j.msard.2019.101897
11. Figueroa-Vargas A, Cárcamo C, Henríquez-Ch R, Zamorano F, Ciampi E, Uribe-San-Martin R, Vásquez M, Aboitiz F, Billeke P: **Frontoparietal connectivity correlates with working memory performance in multiple sclerosis**. *Sci Rep* 2020, **10**(1):9310. **doi**: 10.1038/s41598-020-66279-0
