# Supplementary material for: Functional Connectivity at Rest and During Cognitive Processing in Multiple Sclerosis: A Pilot Magnetoencephalography Study and Systematic Review
Source: Brain Sci. 2026 Aug 14;16(8):862. doi: 10.3390/brainsci16080862 (PMC13511113; doi:10.3390/brainsci16080862)
Supplement: Supplementary file 1 [file brainsci-16-00862-s001.zip › Supplementary tables/Supplementary Table S4, ROB.pdf]

Supplementary Table S4: Risk-of-bias and quality assessment for all 44 included studies, rated using the NIH quality as-sessment tool. Supplementary Figure S1: Resting-state theta band connectivity differences be-tween MS patients and HCs

| Study ID | 1. Was the research question or objective in this paper clearly stated? | 2. Was the study popula-tion clearly specified and defined? | 3. Was the par-ticipation rate of eligible persons at least 50%? | 4. Were all the subjects selected or recruited from the same or similar populations (in-cluding the same time period)? Were inclusion and exclusion crite-ria for being in the study pre-specified and ap-plied uniformly to all participants? | 5. Was a sample size justifica-tion, power de-scription, or variance and ef-fect estimates pro-vided? | 6. For the analyses in this paper, were the expo-sure(s) of in-terest meas-ured prior to the outcome(s) be-ing measured? | 7. Was the time frame sufficient so that one could reasonably ex-pect to see an association between expo-sure and out-come if it ex-isted? | 8. Did the study exam-ine different levels of the exposure (e.g., lesion load, disabil-ity severity)? | 9. Were ex-posure measures (e.g., MS di-agnosis) valid and re-liable? | 10. Was the exposure(s) as-sessed more than once over time? | 11. Were the outcome measures (de-pendent varia-bles) clearly de-fined, valid, reliable, and im-plemented con-sistently across all study partici-pants? | 12. Were the outcome asses-sors blinded to the exposure sta-tus of partici-pants? | 13. Was loss to fol-low-up af-ter base-line 20% or less? | 14. Were key potential con-founding varia-bles measured and adjusted statistically for their impact on the relationship between expo-sure(s) and out-come(s)? | total scores | Quality rating: good (11-14 points) or fair (7.5-10.5 points) or poor (0-7 points) |
|---|---|---|---|---|---|---|---|---|---|---|---|---|---|---|---|---|
| Akbarian 2023 | Yes | Yes | NR | Yes | No | No | No | Yes | Yes | No | Yes | NR | N/A | Yes | 9 | Fair |
| Akbarian 2024 | Yes | Yes | NR | Yes | No | No | No | Yes | Yes | No | Yes | NR | N/A | Yes | 9 | Fair |
| Akbarian 2025 | Yes | Yes | NR | Yes | No | No | No | Yes | Yes | No | Yes | NR | N/A | Yes | 9 | Fair |
| Arpin 2017 | Yes | Yes | NR | Yes | Yes | No | No | Yes | Yes | No | Yes | NR | N/A | Yes | 9.5 | Fair |
| Arpin 2017 (2) | Yes | Yes | NR | Yes | No | No | No | Yes | Yes | No | Yes | NR | N/A | Yes | 9 | Fair |
| Arpin 2018 | Yes | Yes | NR | Yes | No | No | No | Yes | Yes | No | Yes | NR | N/A | Yes | 9 | Fair |
| Barratt 2017 | Yes | Yes | NR | Yes | No | No | No | Yes | Yes | No | Yes | NR | N/A | Yes | 9 | Fair |
| Costers 2020 | Yes | Yes | NR | Yes | No | No | No | Yes | Yes | No | Yes | NR | N/A | Yes | 9 | Fair |
| Cover 2006 | Yes | Yes | NR | Yes | No | No | No | Yes | Yes | No | Yes | Yes | N/A | Yes | 10 | Fair |
| Cipriano 2023 | Yes | Yes | NR | Yes | Yes | No | No | Yes | Yes | No | Yes | NR | N/A | Yes | 9.5 | Fair |
| Cipriano 2024 | Yes | Yes | NR | Yes | No | No | No | Yes | Yes | No | Yes | NR | N/A | Yes | 9 | Fair |
| Dell'Acqua 2009 | Yes | Yes | NR | Yes | No | No | No | Yes | Yes | No | Yes | Yes | N/A | Yes | 10 | Fair |
| Georgopoulos 2007 | Yes | Yes | NR | Yes | No | No | No | No | Yes | No | Yes | NR | N/A | Yes | 8.5 | Fair |
| Hagiwara 2010 | Yes | Yes | NR | Yes | No | No | No | Yes | Yes | No | Yes | NR | N/A | Yes | 9 | Fair |
| Hardmeier 2012 | Yes | Yes | NR | Yes | No | No | No | Yes | Yes | No | Yes | NR | N/A | Yes | 9 | Fair |
| Has Silemek 2023 | Yes | Yes | NR | Yes | No | Yes | Yes | Yes | Yes | Yes | Yes | NR | Yes | Yes | 11.5 | Good |
| Karhu 1992 | Yes | Yes | NR | Yes | No | No | No | No | Yes | No | Yes | NR | N/A | No | 8 | Fair |
| Kassubek 1999 | Yes | Yes | NR | Yes | No | No | No | No | Yes | No | Yes | NR | N/A | No | 8 | Fair |
| Kim 2018 | Yes | Yes | NR | Yes | No | No | No | Yes | Yes | No | Yes | NR | N/A | Yes | 9 | Fair |
| Kim 2020 | Yes | Yes | NR | Yes | No | No | No | Yes | Yes | No | Yes | NR | N/A | Yes | 9 | Fair |
| Kotini 2007 | Yes | Yes | NR | Yes | No | No | No | No | Yes | No | Yes | NR | N/A | No | 8 | Fair |
| Kulik 2021 | Yes | Yes | NR | Yes | No | No | No | Yes | Yes | No | Yes | NR | N/A | Yes | 9 | Fair |
| Mazzara 2025 | Yes | Yes | NR | Yes | No | No | No | Yes | Yes | No | Yes | NR | N/A | Yes | 9 | Fair |
| Nauta 2020 | Yes | Yes | NR | Yes | No | Yes | Yes | Yes | Yes | Yes | Yes | NR | No | Yes | 11 | Good |
| Nauta 2024 | Yes | Yes | NR | Yes | Yes | Yes | Yes | Yes | Yes | Yes | Yes | Yes | Yes | Yes | 13 | Good |
| Nij Bijvank 2021 | Yes | Yes | NR | Yes | No | No | No | Yes | Yes | No | Yes | NR | N/A | Yes | 9 | Fair |
| Paolicelli 2021 | Yes | Yes | NR | Yes | No | No | No | Yes | Yes | No | Yes | NR | N/A | Yes | 9 | Fair |
| Rossi 2024 | Yes | Yes | NR | Yes | No | No | No | Yes | Yes | No | Yes | NR | N/A | Yes | 9 | Fair |
| Sanders 2025 | Yes | Yes | NR | Yes | No | No | No | Yes | Yes | No | Yes | NR | N/A | Yes | 9 | Fair |
| Schoonheim 2013 | Yes | Yes | NR | Yes | No | No | No | Yes | Yes | No | Yes | NR | N/A | Yes | 9 | Fair |
| Schoonhoven 2019 | Yes | Yes | NR | Yes | No | No | No | Yes | Yes | No | Yes | Yes | N/A | Yes | 10 | Fair |

| | | | | | | | | | | | | | | | | |
|---|---|---|---|---|---|---|---|---|---|---|---|---|---|---|---|---|
| **Simon 2023** | Yes | Yes | NR | Yes | No | No | No | Yes | Yes | No | Yes | Yes | N/A | Yes | 10 | Fair |
| **Sjøgård 2020** | Yes | Yes | NR | Yes | No | No | No | Yes | Yes | No | Yes | NR | N/A | Yes | 9 | Fair |
| **Sorrentino 2024** | Yes | Yes | NR | Yes | No | No | No | Yes | Yes | No | Yes | NR | N/A | Yes | 9 | Fair |
| **Stickland 2019** | Yes | Yes | NR | Yes | No | No | No | Yes | Yes | No | Yes | NR | N/A | Yes | 9 | Fair |
| **Tewarie 2013** | Yes | Yes | NR | Yes | No | No | No | Yes | Yes | No | Yes | NR | N/A | Yes | 9 | Fair |
| **Tewarie 2014** | Yes | Yes | NR | Yes | No | No | No | Yes | Yes | No | Yes | NR | N/A | Yes | 9 | Fair |
| **Tewarie 2014 (1)** | Yes | Yes | NR | Yes | No | No | No | Yes | Yes | No | Yes | NR | N/A | Yes | 9 | Fair |
| **Tewarie 2014 (2)** | Yes | Yes | NR | Yes | No | No | No | Yes | Yes | No | Yes | NR | N/A | Yes | 9 | Fair |
| **Tewarie 2017** | Yes | Yes | NR | Yes | No | No | No | Yes | Yes | No | Yes | NR | N/A | Yes | 9 | Fair |
| **Van der meer 2013** | Yes | Yes | NR | Yes | No | No | No | Yes | Yes | No | Yes | NR | N/A | Yes | 9 | Fair |
| **Van Schependom 2019** | Yes | Yes | NR | Yes | No | No | No | Yes | Yes | No | Yes | NR | N/A | Yes | 9 | Fair |
| **Van Schependom 2021** | Yes | Yes | NR | Yes | No | No | No | Yes | Yes | No | Yes | NR | N/A | Yes | 9 | Fair |
| **Waldman 2020** | Yes | Yes | NR | Yes | No | No | No | Yes | Yes | No | Yes | NR | N/A | Yes | 9 | Fair |
